# Understanding the biosynthesis, metabolic regulation, and anti-phytopathogen activity of 3,7-dihydroxytropolone in *Pseudomonas* spp.

**DOI:** 10.1128/mbio.01022-24

**Published:** 2024-08-29

**Authors:** Alaster D. Moffat, Lars Höing, Javier Santos-Aberturas, Tim Markwalder, Jacob G. Malone, Robin Teufel, Andrew W. Truman

**Affiliations:** 1 Department of Molecular Microbiology, John Innes Centre, Norwich, United Kingdom; 2 Department of Pharmaceutical Sciences, University of Basel, Basel, Switzerland; 3 School of Biological Sciences, University of East Anglia, Norwich, United Kingdom; McMaster University, Hamilton, Ontario, Canada

**Keywords:** natural antimicrobial products, biosynthesis, *Pseudomonas*, specialized metabolism, biosynthetic gene cluster

## Abstract

The genus *Pseudomonas* is a prolific source of specialized metabolites with significant biological activities, including siderophores, antibiotics, and plant hormones. These molecules play pivotal roles in environmental interactions, influencing pathogenicity, inhibiting microorganisms, responding to nutrient limitation and abiotic challenges, and regulating plant growth. These properties mean that pseudomonads are suitable candidates as biological control agents against plant pathogens. Multiple transposon-based screens have identified a *Pseudomonas* biosynthetic gene cluster (BGC) associated with potent antibacterial and antifungal activities, which produces 7-hydroxytropolone (7-HT). In this study, we show that this BGC also makes 3,7-dihydroxytropolone (3,7-dHT), which has strong antimicrobial activity toward *Streptomyces scabies*, a potato pathogen. Through metabolomics and reporter assays, we unveil the involvement of cluster-situated genes in generating phenylacetyl-coenzyme A, a key precursor for tropolone biosynthesis via the phenylacetic acid catabolon. The clustering of these phenylacetic acid genes within tropolone BGCs is unusual in other Gram-negative bacteria. Our findings support the interception of phenylacetic acid catabolism via an enoyl-CoA dehydratase encoded in the BGC, as well as highlighting an essential role for a conserved thioesterase in biosynthesis. Biochemical assays were used to show that this thioesterase functions after a dehydrogenation–epoxidation step catalyzed by a flavoprotein. We use this information to identify diverse uncharacterized BGCs that encode proteins with homology to flavoproteins and thioesterases involved in tropolone biosynthesis. This study provides insights into tropolone biosynthesis in *Pseudomonas*, laying the foundation for further investigations into the ecological role of tropolone production.

**IMPORTANCE:**

*Pseudomonas* bacteria produce various potent chemicals that influence interactions in nature, such as metal-binding molecules, antibiotics, or plant hormones. This ability to synthesize bioactive molecules means that *Pseudomonas* bacteria may be useful as biological control agents to protect plants from agricultural pathogens, as well as a source of antibiotic candidates. We have identified a plant-associated *Pseudomonas* strain that can produce 3,7-dihydroxytropolone, which has broad biological activity and can inhibit the growth of *Streptomyces scabies*, a bacterium that causes potato scab. Following the identification of this molecule, we used a combination of genetic, chemical, and biochemical experiments to identify key steps in the production of tropolones in *Pseudomonas* species. Understanding this biosynthetic process led to the discovery of an array of diverse pathways that we predict will produce new tropolone-like molecules. This work should also help us shed light on the natural function of antibiotics in nature.

## INTRODUCTION

The bacterial genus *Pseudomonas* is widely distributed in nature, where it is ubiquitously found in soils worldwide (1), with extraordinary inter-species and intra-species diversity that is reflected in a pangenome of over 60,000 unique genes (2, 3). As a result of this tremendous diversity, *Pseudomonas* spp. are a well-known source of novel specialized metabolites (SMs) with important biological activities (4–6), including siderophores like pyoverdine and pyochelin (7), antibiotics such as obafluorin (8) and mupirocin (9), extracellular electron shuttles such as phenazines (10), as well as a diverse repertoire of volatile compounds (11). *Pseudomonas* SMs often have critical roles in mediating interactions with other organisms in the environment (12–14), including toxins associated with pathogenicity, antibiotics and antifungals to inhibit other microorganisms, and hormones that influence plant growth and development (12). The capacity of *Pseudomonas* spp. to colonize plant roots and inhibit other organisms means that many strains of this genus have already been targeted for use in crop protection programs as biological control agents that can suppress plant pathogens (15).

A significant number of SM biosynthetic gene clusters (BGCs) do not possess canonical features such as polyketide synthases (PKSs) or non-ribosomal peptide synthetases (NRPSs), which enable detection by widely used genome mining tools and are instead discovered using activity-guided transposon mutagenesis screens. Recently, several independent high-throughput transposon-based screens discovered a *Pseudomonas* BGC associated with potent antibacterial and antifungal activities, including our own work, which shows its involvement in the inhibition of *Streptomyces scabies*, a bacterium that causes potato scab, by *Pseudomonas* sp. Ps652 (16). This BGC was initially identified by Xie and co-workers using a transposon screen of *Pseudomonas donghuensis* HYS to identify the BGC of a non-fluorescent siderophore (17), which was later determined to be 7-hydroxytropolone (7-HT) (18, 19). This tropolone BGC was also independently identified by transposon mutagenesis as the key determinant of the suppressive activity of *P. donghuensis* isolates toward the bacterium *Pectobacterium carotovorum* (20) (potato black leg and soft rot) and the fungus *Macrophomina phaseolina* (21) (broad-spectrum stem and root rot), as well as the fungus *Verticillium dahliae* (cotton verticillium wilt) via targeted inactivation of the BGC (22).

**Fig 1:**
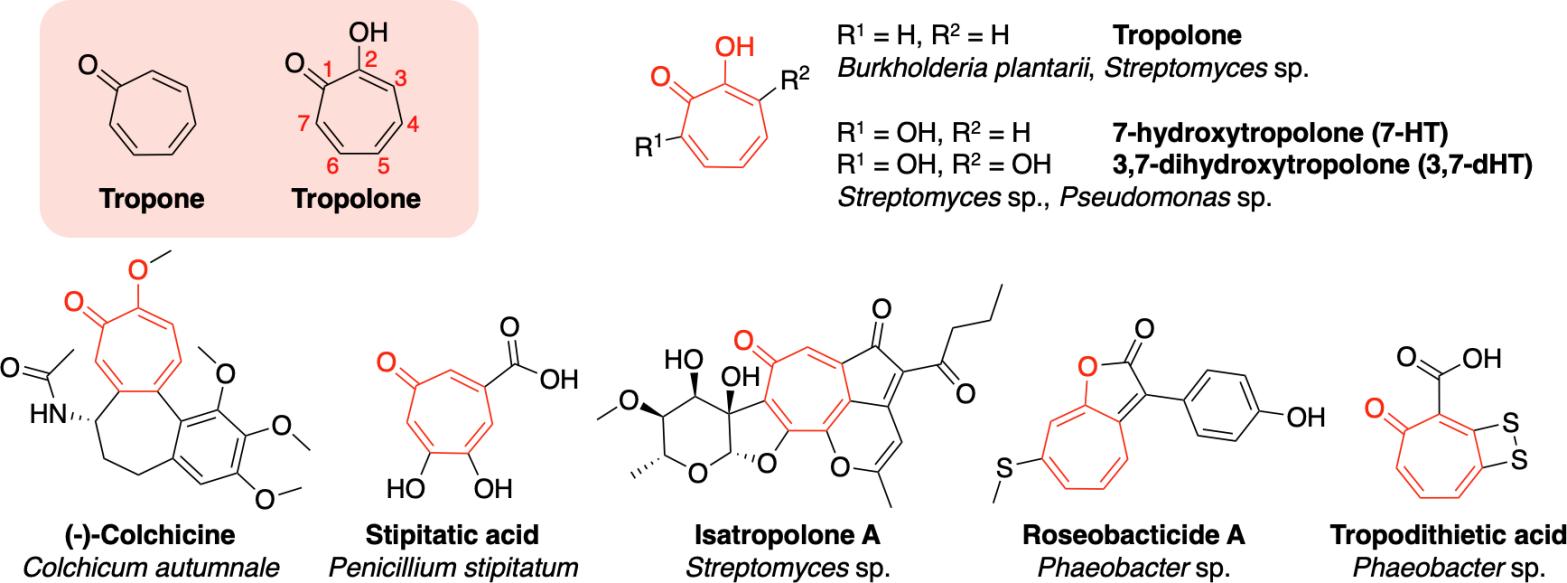
The tropone and tropolone moieties alongside examples of tropolone natural products and their diverse bacterial, fungal, and plant producers.

Tropolones are small molecules containing an aromatic 7-membered cyclohepta-2,4,6-trienone (tropone) ring that is hydroxylated at position 2 (Fig. 1). Tropolone SMs have been documented in plants, fungi, and bacteria and possess an extensive range of biological activities, including antibacterial, antifungal, antiviral, and cytotoxic activities (23, 24). The diverse origins of tropolone-containing SMs mean that the tropolone moiety can be produced via a variety of distinct biosynthetic routes, such as via PKSs in the biosynthesis of stipitatic acid (25) and isatropolone (26) (Fig. 1), or from a tyrosine-derived moiety that has undergone ring expansion in colchicine biosynthesis (27). In bacteria, tropolone biosynthesis has been a long-standing question since the first discovery of tropolone production by *Pseudomonas* ATCC 31099 in 1980 (28). The genetic evidence for the route to the standalone tropolone skeleton was first determined via transposon mutagenesis to identify genes in Rhodobacteraceae involved in the biosynthesis of tropodithietic acid, a sulfur-containing tropone (29).

Further studies demonstrated that tropodithietic acid is derived from phenylacetic acid (PAA) catabolism (30, 31) via a key branching point (32–35) to the direct flux of primary metabolic intermediates toward the biosynthesis of a 7-membered ring tropolone precursor, which was first proposed in 1992 following isotope feeding experiments on thiotropocin biosynthesis (36). A conceptually similar pathway occurs in *Streptomyces* (37, 38), which also branches from the PAA degradative pathway, followed by additional modifications by dedicated biosynthetic enzymes, but this BGC appears to be evolutionarily distinct to the Gram-negative tropolone BGCs found in *Phaeobacter*, *Burkholderia,* and *Pseudomonas*. We recently described a novel class of dioxygenase in *Burkholderia* capable of producing tropolone via the PAA catabolon (39), but questions still remain about earlier biosynthetic steps, the full complement of genes required for the production of tropolones, and the function of enzymes catalyzing later-stage modifications, such as additional hydroxylation or incorporation of sulfur atoms. In particular, very little is known about tropolone biosynthesis in *Pseudomonas*.

In this study, we report that the tropolone (*tpo*) BGC in *Pseudomonas* sp. Ps652 is responsible for the production of 3,7-dihydroxytropolone (3,7-dHT), which may represent the true product of the *tpo* BGC. This is the first time that 3,7-dHT production has been reported for a Gram-negative bacterium. This metabolite is a critical determinant of suppressive activity toward the potato pathogens *S. scabies*, a bacterium that causes common scab, and *Phytophthora infestans*, an oomycete that causes potato late blight. We show that 3,7-dHT has greater potency against *S. scabies* 87–22 when compared to 7-HT and investigate the role of a conserved thiamine pyrophosphate (TPP)-dependent decarboxylase and a phenylacetyl-coenzyme A (PA-CoA) ligase in the early stages of biosynthesis and regulation of the pathway. These two genes form part of the core BGC, serving to derepress the PAA catabolon under conditions of low environmental PAA availability, thereby allowing production of tropolones in those conditions. We also demonstrate that a conserved thioesterase has a key role alongside a flavoprotein for a late-stage step in tropolone biosynthesis and use this information to identify new BGCs.

## RESULTS

### Gene deletions highlight the importance of tropolone production for suppressive activity

Our previous transposon mutagenesis study (16) highlighted an important role for the *tpo* BGC in the inhibition of *S. scabies* by *Pseudomonas* sp. Ps652 (simply referred to as Ps652), an environmental isolate from a commercial potato field in Norfolk (U.K.). However, that transposon screen may have missed the contributions of the other BGCs, especially toward other plant pathogens that were not part of the screen, or if a BGC was too small to be frequently hit via transposon mutagenesis. To ensure that BGCs were accurately annotated, we obtained a higher-quality single-scaffold genome assembly, produced by Illumina and Oxford Nanopore sequencing (accession OZ024668.1), that improved on the previous 80 contig assembly. Multilocus sequence typing (MLST) using AutoMLST (40) (Fig. S1) showed that Ps652 possesses 93.5% average nucleotide identity (ANI) to *Pseudomonas* sp. UC 17F4 (GCF_900101695) and 93.4% to *Pseudomonas donghuensis* (GCF_000259195), a previously characterized tropolone producer (19). These ANIs fall below the 94.0% threshold set for the distinction of species in *Pseudomonas* (41, 42). Accordingly, it is likely that Ps652 represents a novel species of *Pseudomonas*, which is also supported by an analysis using the Type Strain Genome Server (43). Phylogenetic analysis places it within the *P. vranovensis* sub-group within the *P. putida* group (44) (Fig. S1).

**Fig 2:**
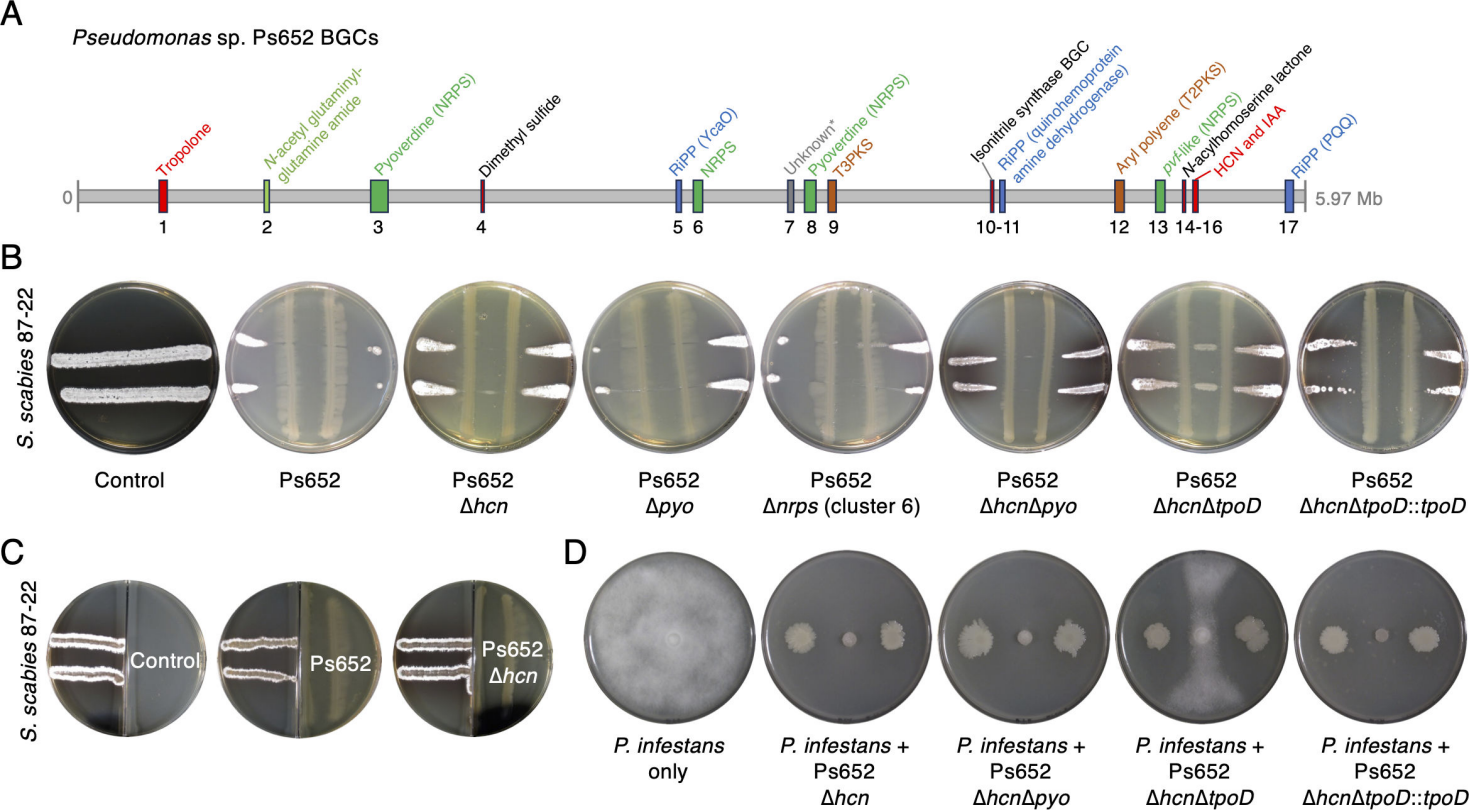
Ps652 BGCs and the effect of deletions on the suppressive activity. (A) Overview of BGCs identified from antiSMASH 7.0, GECCO 0.9.8, and manual annotation. *Unknown relates to a GECCO hit with a low number of biosynthetic genes. Table S5 provides further BGC details. (B) Cross-streak assays between Ps652 mutants and *S. scabies* showing qualitative inhibitory activity. Control = *S. scabies* only without addition of Ps652. *S. scabies* was able to grow between the Ps652 streaks in the ∆*hcn*∆*tpoD* mutant, but not in other mutants. (C) Split-plate assays assessing the role of volatiles in *S. scabies* inhibition. No change in the *S. scabies* morphology (left hand side) was detected with either Ps652 or the *hcn* deletion mutant. (D) Assays between *P. infestans* (central growth) and Ps652 mutants. Further split-plate assays are shown in Fig. S2.

The BGC detection tool antiSMASH 7.0.0 (45) detected 10 BGCs in the Ps652 genome (Fig. 2A; Table S5), including BGCs predicted to be involved in the biosynthesis of the siderophore pyoverdine, an aryl polyene (46); the redox cofactor pyrroloquinoline quinone (47) (PQQ); and a homolog of the *Pseudomonas virulence factor* (*pvf*) BGC, which is predicted to produce autoinducers that regulate gene expression (48). Analysis using GECCO, an alternative BGC identification tool (49), revealed two more putative BGCs (Table S5), but neither tool detected the tropolone BGC. As reported previously (13), further analysis of the genome revealed genes for the biosynthesis of hydrogen cyanide (50) (HCN), hydrogen sulfide (51), and indole-3-acetic acid (52) (IAA), a common plant auxin (Fig. 2A).

HCN is known to have an inhibitory effect toward some plant pathogens (50), but deletion of the HCN BGC had a negligible effect on biological activity toward *S. scabies* (Fig. 2B) and *P. infestans* (Fig. 2D). No other BGCs were predicted to produce antimicrobial molecules, so in-frame deletions were made in a BGC encoding a standalone NRPS module with an unknown function (∆*nrps*, BGC6, Fig. 2A; Table S5) and in the pyoverdine BGC (∆*pyo*) to assess whether iron chelation by pyoverdine contributed to suppressive activity. Neither of these mutants altered the suppressive activity. Split-plate assays using plates with an internal barrier (Fig. 2C) indicated that volatiles were not major determinants of suppression toward either pathogen, although volatile activity was medium-dependent for *P. infestans* inhibition (Fig. S2), highlighting the production of further suppressive molecules.

**Fig 3:**
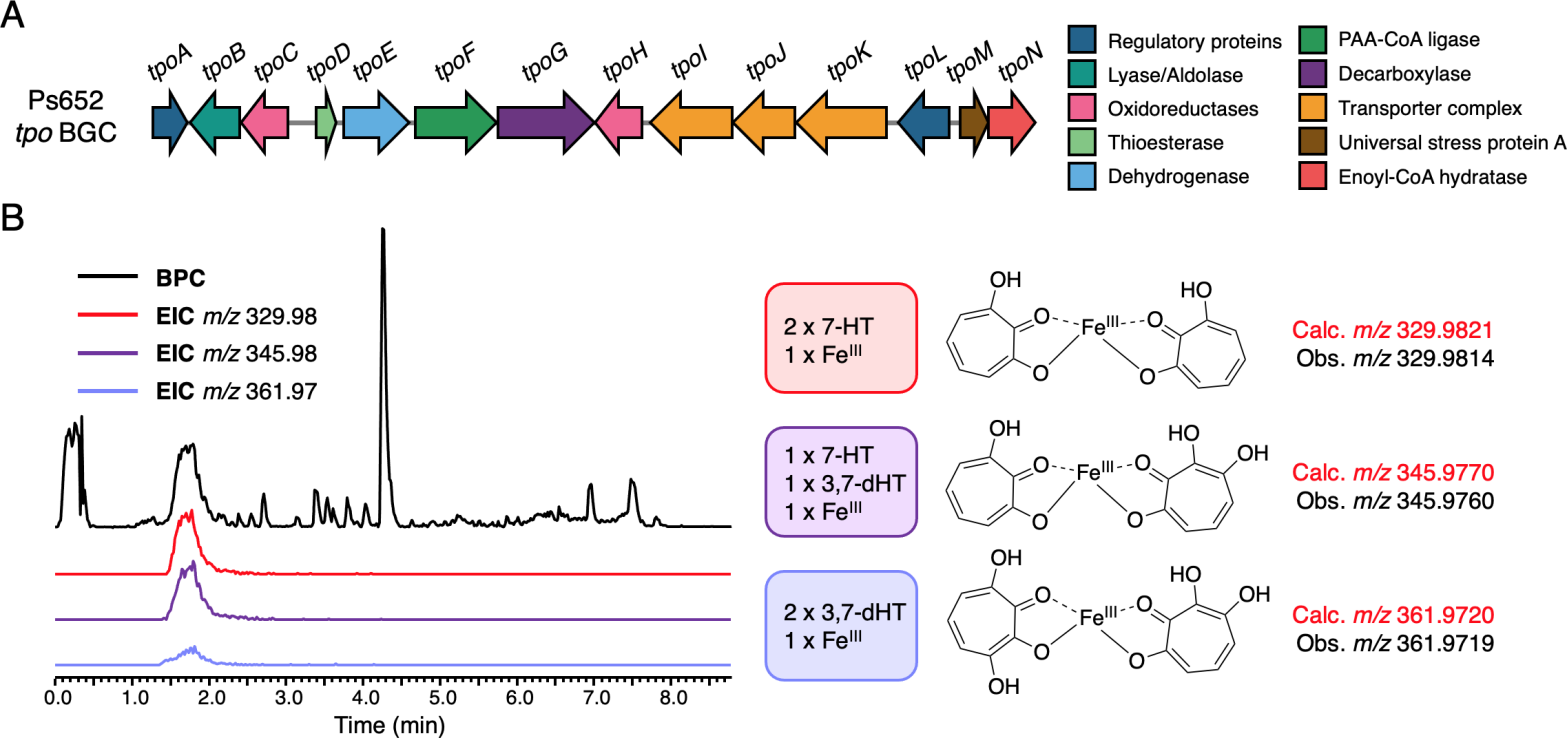
Tropolone biosynthesis in Ps652. (A) The tropolone BGC in *Pseudomonas* sp. Ps652 and a new suggested naming scheme. (B) Tropolone–iron chelates observed in LC-MS data of extracts from Ps652 ∆*hcn* that suggested the production of an additional dihydroxylated tropolone (BPC = base peak chromatogram; EIC = extracted ion chromatogram).

A gene naming scheme for the *Pseudomonas* tropolone BGC (*tpo*) is proposed based on BGC boundaries inferred from mutants generated in this study and prior studies on homologous BGCs in other *Pseudomonas* strains (16, 17, 19, 21, 22) (Fig. 3A; Table S6). To support the role of the *tpo* BGC in *S. scabies* inhibition and to rule out the possibility that prior transposon mutants had unintended polar effects on Ps652, an in-frame deletion was generated in *tpoD*, which was hit twice in prior transposon mutagenesis work (16). All mutants were generated in Ps652 ∆*hcn* to eliminate the possibility that HCN-based inhibition obscured *tpo*-dependent effects in subsequent assays. *tpoD* encodes a predicted acyl-CoA thioesterase, and homologs are conserved across previously identified tropolone BGCs in Gram-negative bacteria but without a documented role to date. This ∆*hcn*∆*tpoD* mutant resulted in the loss of suppression of *S. scabies* 87–22 growth at a distance, where *S. scabies* growth could be observed between the Ps652 streaks (Fig. 2B) and could be genetically complemented by the expression of *tpoD* from plasmid pME6032 (53) (Fig. 2B). Suppressive activity toward *P. infestans* was significantly reduced in Ps652 ∆*hcn*∆*tpoD,* but not completely abolished, indicating that the *tpo* BGC contributes to suppression but that there are further anti-oomycete SMs produced by Ps652 (Fig. 2D).

### *Pseudomonas* sp. Ps652 produces multiple tropolones

**Fig 4:**
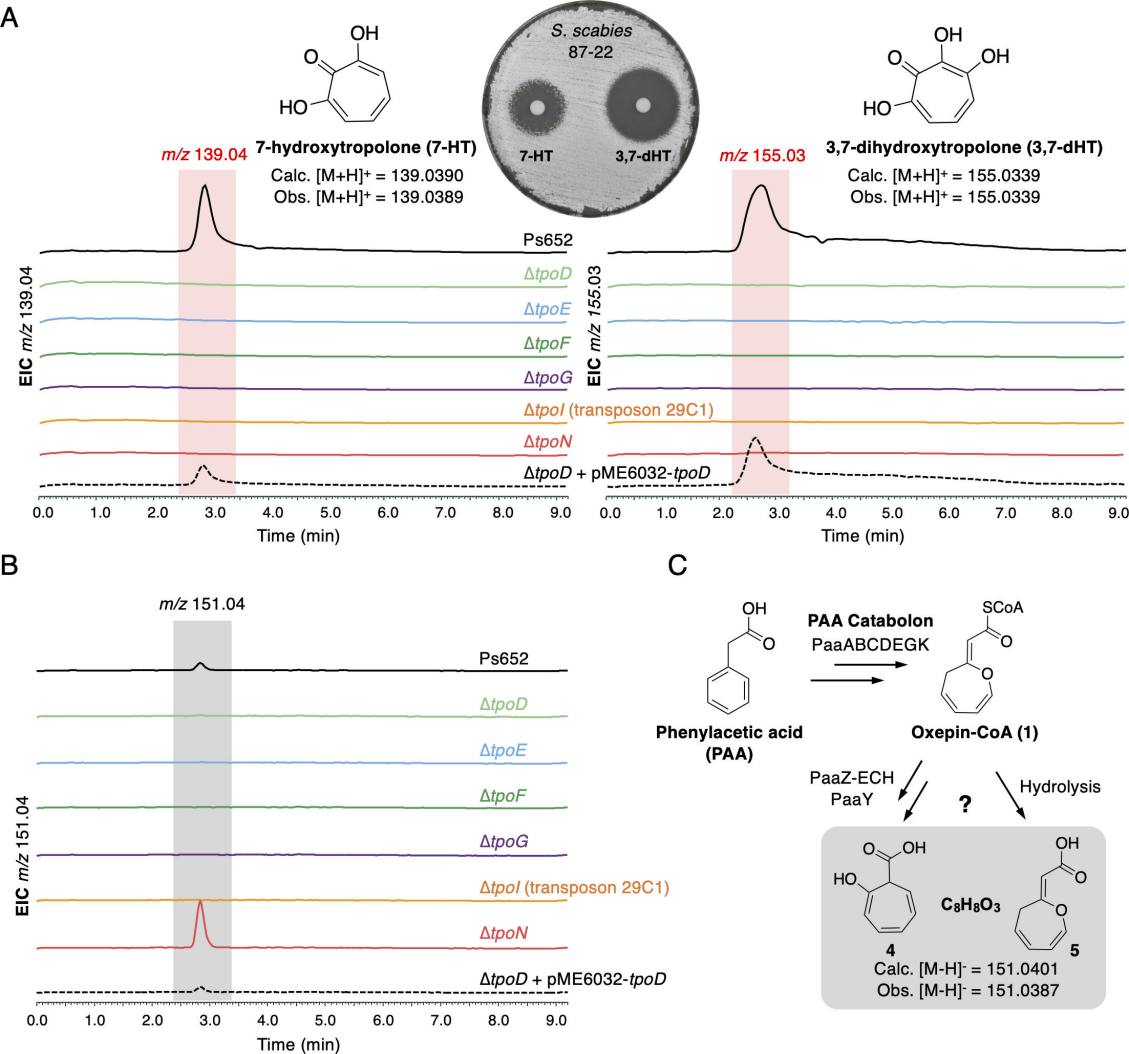
Gene deletions in the Ps652 tropolone BGC. A. LC-MS spectra showing production of 7-HT and 3,7-HT by Ps652. Deletion of *tpoD* abolishes the production of tropolones, which can be restored by genetic complementation with pME6032-*tpoD*. Production is also abolished across each tropolone BGC mutant. Each mutant is generated in Ps652 ∆*hcn*. 3,7-dHT is more active against *S. scabies* compared to 7-HT (10 µg of each compound used in assay). B. Production of *m/z* 151.04 molecule (negative-mode MS) across BGC mutants. C. Routes to two possible shunt metabolites that have a mass consistent with that of the observed molecule.

After discovery of the tropolone BGC in Ps652 and confirmation of its link to suppressive activity toward *S. scabies* 87–22 and *P. infestans*, it was essential to establish whether Ps652 indeed produces tropolones. Using a synthetic standard of 7-HT for comparison in liquid chromatography–mass spectrometry (LC-MS) analyses, we were able to detect 7-HT (calculated [M + H]^+^ *m/z* 139.0390; observed *m/z* 139.0389) in extracts of Ps652 (Fig. S3). 7-HT was undetectable in the equivalent extracts from Ps652 ∆*hcn*∆*tpoD*, confirming its identity as a hydroxylated tropolone related to the BGC. However, we also observed some molecules with the characteristic 330 nm absorbance of tropolones but with larger mass-to-charge ratios (Fig. 3B; Fig. S3) that were consistent with the masses of tropolone–iron chelates, matching the 2:1 tropolone to iron stoichiometry demonstrated by Jiang *et al* (18). Interestingly, some of these masses corresponded to the predicted masses of a dihydroxylated tropolone in complex with iron and 7-HT (calculated *m/z* 345.9770; observed *m/z* 345.9760) or two molecules of a dihydroxylated tropolone in complex with iron(III) (calculated *m/z* 361.9718; observed *m/z* 361.9719) (Fig. S3). The corresponding monomer of the dihydroxylated tropolone was subsequently detected in MS data ([M + H]^+^, calculated *m/z* 155.0339; observed *m/z* 155.0339) (Fig. 4A; Fig. S4).

We purified this dihydroxylated tropolone from Ps652 ∆*hcn*∆*pyo* to yield 2.8 mg of pure material. NMR analysis (Fig. S5 and S6) identified the molecule as 3,7-dihydroxytropolone (3,7-dHT), matching a previously published characterization (54) with ∂_C_ = 119.2 ppm, 129.4 ppm, 158.1 ppm, and 158.9 ppm. 3,7-dHT has the ability to bind iron equivalent to or greater than 7-HT in a qualitative assay on chrome azurol S agar (55) (Fig. S7), suggesting it is also capable of functioning as a siderophore, like 7-HT (18). Additionally, disk diffusion assays against *S. scabies* 87–22 indicated that 3,7-dHT is a more potent antibiotic compared to 7-HT (Fig. 4A), with respective minimum inhibitory concentrations (MICs) of 2.5 and 5 µg/mL (Fig. S7). Together, these data suggest that 3,7-dHT may constitute the true final product of the tropolone BGC in *Pseudomonas*, whereas 7-HT could be a biosynthetic intermediate or shunt metabolite lacking the final hydroxylation. No additionally hydroxylated tropolones were observed in our LC-MS data.

### Understanding tropolone biosynthesis in *Pseudomonas*

In order to understand which genes are genuinely required for tropolone biosynthesis, we generated a series of nonpolar deletion mutants to supplement previously generated transposon mutants, which included *tpoI* (encoding a putative transporter component; transposon mutant 29C1). In addition to *tpoD* (encoding a putative thioesterase), in-frame mutants were also generated in Ps652 ∆*hcn* for genes encoding TpoE (dehydrogenase-like flavoprotein), TpoF (acyl-CoA ligase), TpoG (TPP-dependent decarboxylase), and TpoN (enoyl-CoA dehydratase). All mutants were unable to produce 7-HT and 3,7-dHT, demonstrating the involvement of the deleted genes in tropolone biosynthesis (Fig. 4A). Genetic complementation of *tpoE* and *tpoF* using pUC18-mini-Tn7-based plasmids (56) restored tropolone biosynthesis (Fig. S8), indicating that there were no unanticipated secondary effects of deleting these mid-operon genes.

**Fig 5:**
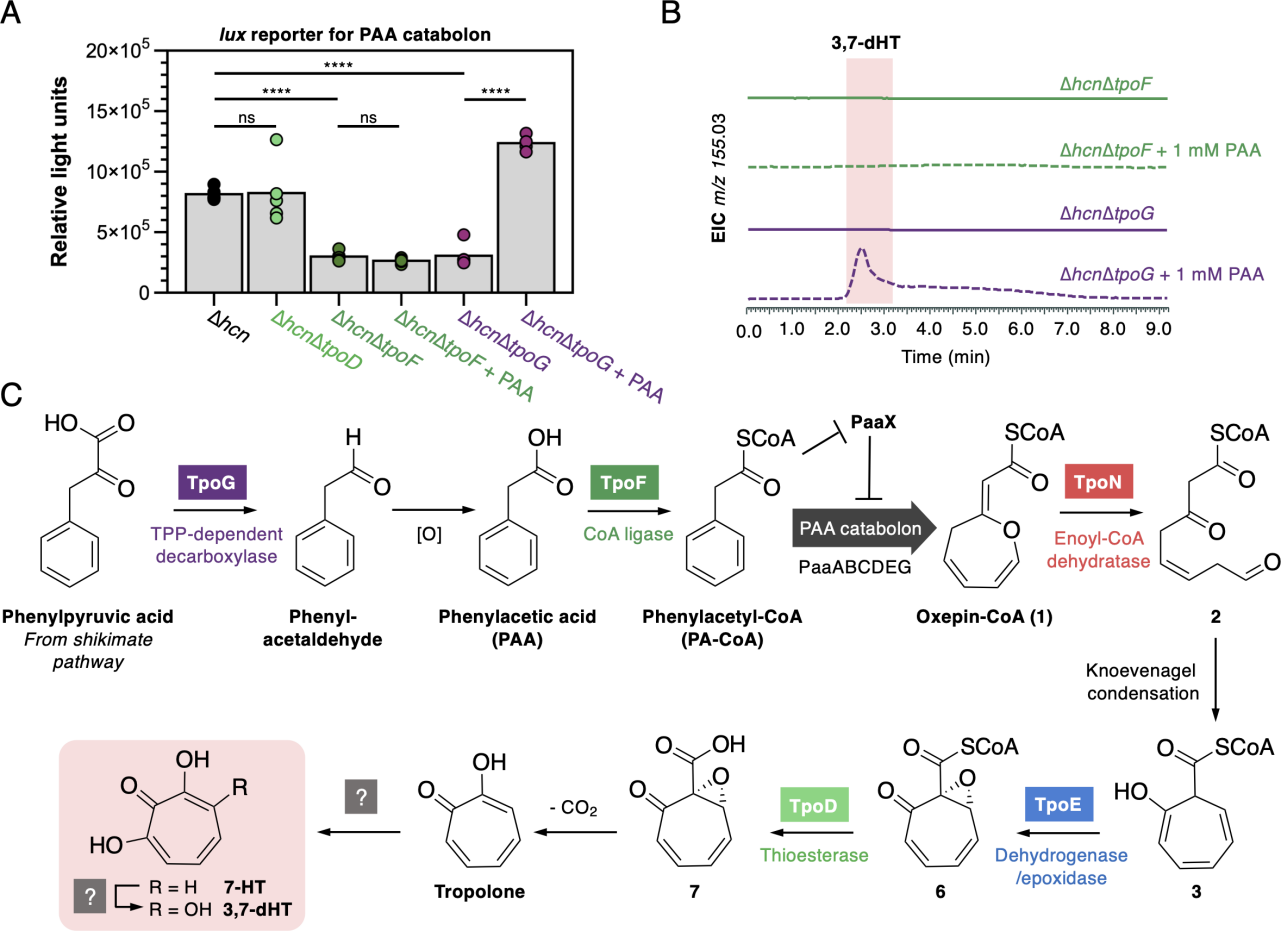
Role of TpoF and TpoG in tropolone biosynthesis. (A) Luciferase (*lux*) reporter assay for the expression of the PAA catabolon in different Ps652 mutant strains. PAA was added at a concentration of 1 mM. Data were collected for five replicates for each condition, which are presented along with the respective means (gray bars). Significance determined by Student’s *t*-test (two-tailed, unequal variance) is presented: ns = not significant (*P* > 0.05), **** =*P* < .0001. (B) LC-MS spectra showing the chemical complementation of ∆*tpoG* with exogenous PAA, whereas production is not restored when PAA is fed to ∆*tpoF*. (C) Proposed biosynthetic scheme for 7-HT and 3,7-dHT in Ps652.

However, we were unable to identify intermediates or shunt metabolites from most mutants via targeted and untargeted analyses of LC-MS spectra. The lack of detectable molecules could be caused by compound instability, volatility, and/or the disruption of early biosynthetic steps. One exception was ∆*tpoN*, where a molecule of *m/z* 151.04 was observed in negative-mode MS. This compound was detected in extracts of parental Ps652 ∆*hcn*, but in substantially lower amounts than in ∆*tpoN* (Fig. 4B). TpoN is an enoyl-CoA hydratase (ECH), which is one of the domains of PaaZ, a dual domain protein involved in PAA catabolism that features an ECH domain and an aldehyde dehydrogenase (ALDH) domain. PaaZ catalyzes the ring opening and oxidation of (*Z*)−2-(oxepin-2(3*H*)-ylidene)-acetyl CoA (oxepin-CoA, **1**), a key intermediate in the PAA catabolon. In the absence of a functional ALDH domain, the PaaZ ECH domain catalyzes the formation of a highly reactive 3-oxo-5,6-dehydrosuberoyl-CoA semialdehyde (**2**) that can undergo a spontaneous Knoevenagel condensation to generate a 7-membered tropolone precursor (2-hydroxycyclohepta-1,4,6-triene-1-formyl-CoA, **3**) (32, 57) (Fig. 5).

The instability of the *m/z* 151.04 molecule prevented full characterization, but the accurate mass is consistent with that of 2-hydroxycyclohepta-1,4,6-triene-1-carboxylic acid (**4**) (C_8_H_8_O_3_, calculated [M-H]^-^ *m/z* 151.0401; observed *m/z* 151.0387), which is a shunt metabolite that can be formed by the PAA catabolon via the thioesterase PaaY (Fig. 4C). Alternatively, thioester hydrolysis of **1** would also provide a C_8_H_8_O_3_ molecule (**5**), which could occur if the PAA catabolon stalls prior to PaaZ/TpoN activity (Fig. 4C). In *Streptomyces cyaneofuscatus*, a standalone ECH is proposed to direct PAA flux toward tropolone biosynthesis (37), and our data support an equivalent function for TpoN in *Pseudomonas* given the production of a putative shunt metabolite from the PAA catabolon.

### Role of phenylacetate-CoA ligase (TpoF) and a TPP-dependent decarboxylase (TpoG) in tropolone biosynthesis in *Pseudomonas*

Gene deletions in Ps652 highlighted an essential role for *tpoF* and *tpoG* in tropolone biosynthesis. Prior transposon mutagenesis and gene regulation studies have reported *tpoF* and *tpoG* homologs as important for biological activity and siderophore phenotypes related to tropolone biosynthesis in various *Pseudomonas* strains (17, 19, 21). However, to date, no precise biosynthetic roles have been proposed and homologs of these genes are not found in tropolone BGCs in other Gram-negative bacteria. TpoF is a homolog of the phenylacetyl-CoA (PA-CoA) ligase PaaK from the PAA catabolon. It has been shown in *Escherichia coli* that *paaK* and the rest of the PAA catabolon is only expressed in the presence of PA-CoA, which directly depresses PaaX, a transcriptional repressor that controls the PAA catabolon (57). TpoG is a putative TPP-dependent decarboxylase (Table S6), which is of the same protein family (COG3961) as IpdC from *Azospirillum brasilense*. IpdC catalyzes the decarboxylation of phenylpyruvate to phenylacetaldehyde as part of auxin biosynthesis (58, 59). Phenylacetaldehyde could then be further oxidized to PAA, potentially via a non-clustered phenylacetaldehyde dehydrogenase [Ps652 VVM71318.1 is 52% identical to phenylacetaldehyde dehydrogenase from the *Pseudomonas putida* S12 styrene catabolic pathway (60)]. We therefore hypothesized that, in the absence of PAA or PA-CoA from primary metabolism, TpoG and TpoF would cooperate to supply PA-CoA as a tropolone precursor and to activate the expression of the PAA catabolon, which is required for tropolone production.

To test this hypothesis, a PAA catabolon reporter was generated by fusing the promoter of *paaA* from Ps652 (Fig. S9) to the luciferase (*lux*) operon, which generates bioluminescence when expressed (61). Use of this *paa* promoter*–lux* fusion reporter in ∆*tpoF* and ∆*tpoG* indicated that expression of the PAA catabolon was significantly lower than in the parental Ps652 ∆*hcn* strain (Fig. 5A). Additionally, we observed that supplying exogenous PAA was not sufficient to chemically complement the ∆*tpoF* mutant, but PAA could complement the ∆*tpoG* mutant (Fig. 5B). These results are consistent with TpoG directly increasing the production of PAA and TpoF then converting PAA to PA-CoA. PA-CoA could then function to derepress the PAA catabolon by rendering PaaX inactive. When exogenous PAA is added, PA-CoA can be produced by TpoF in the ∆*tpoG* mutant, but not in the ∆*tpoF* mutant. TpoF and TpoG thus function to supply an essential precursor and activate the PAA catabolon for tropolone production in the absence of environmental PAA (Fig. 5C). A similar situation is seen in the biosynthesis of tropolones in *S. cyaneofuscatus*, where TrlB and TrlH help shuttle primary metabolites toward the PAA catabolon and ultimately tropolone biosynthesis (37), although these proteins have different catalytic roles to TpoF and TpoG.

### TpoD and TpoE function to generate an advanced tropolone precursor

**Fig 6:**
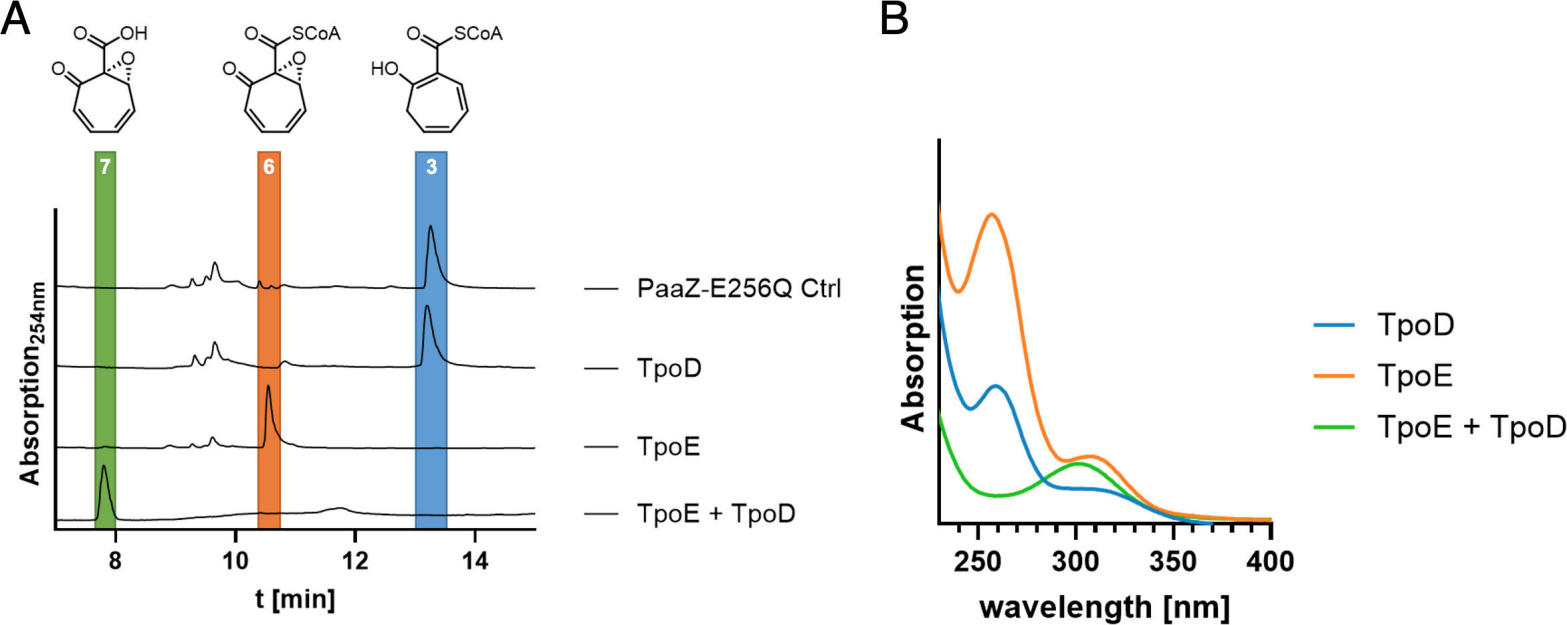
HPLC analysis of biochemical assays with TpoD and TpoE. (A) The PaaZ-E256Q control (Crtl) variant with deficient aldehyde dehydrogenase domain was used to generate 3-oxo-5,6-dehydrosuberoyl-CoA semialdehyde, which undergoes Knoevenagel condensation to yield **3**, highlighted in blue. This precursor was incubated for 15 minutes with 1 µM of TpoD and/or TpoE. For the traces PaaZ-E256Q Ctrl, TpoD, and TpoE, the aqueous phase is shown (no significant amounts of any intermediate could be observed in the organic phase; not shown), for trace TpoE +TpoD, the organic phase is shown (no significant amounts of any intermediate could be observed in the aqueous phase; not shown). (B) UV–visible spectra of the highlighted compounds showing the absence of coenzyme A absorbance at 260 nm for compound **7**.

TpoD (thioesterase) and TpoE (flavoprotein) encoded in the *Pseudomonas tpo* BGC are essential for tropolone biosynthesis (Fig. 4A). TpoE is a homolog of flavoproteins previously shown to be critical for tropodithietic acid biosynthesis in *Phaeobacter inhibens* (TdaE^Pi^) and tropolone biosynthesis in *Burkholderia plantarii* (TdaE^Bp^) (39). TdaE^Pi^ and TdaE^Bp^ homologs were furthermore shown to convert **3** into (2*R*,3*R*)-epoxytropone-2-carboxylate (**7**, Fig. 5C) and thus function as unusual flavoprotein dioxygenases that mediate ring dehydrogenation, CoA-ester oxygenolysis, and a final stereoselective ring epoxidation (39). To examine the roles of TpoE and TpoD in the biosynthesis of 3,7-dHT in *Pseudomonas* spp., we heterologously produced and purified these enzymes. Similarly, enzymes from the early PAA catabolism (PaaABCE, PaaG, and PaaZ-E256Q) were obtained and employed for the biochemical generation of **3** (30, 32, 34), which represents the likely substrate for TpoE. Following enzymatic formation of **3** (39), the assays were complemented by TpoE, TpoD, or their combination. Interestingly, while the addition of TpoD had no effect, TpoE converted **3** into a new compound, whose mass and spectroscopic properties were consistent with the formation of epoxytropone-2-carboxyl-CoA **6**, resulting from ring dehydrogenation and subsequent epoxidation (Fig. 6; Fig. S10). Surprisingly, in contrast to previous observations with the homolog TdaE^Pi^ from *P. inhibens* (39), TpoE appeared not to cleave the thioester itself. This implied the necessity for an additional thioesterase functionality en route to 3,7-dHT, for which TpoD is an evident prime candidate. Indeed, when both enzymes were added together, **7** was produced, as verified by LC-MS analysis and compared to the identical TdaE-produced compound (Fig. S10 and S11). Taken together, these assays establish the roles of both TpoD and TpoE by showing that they jointly produce the highly reactive **7**, which subsequently degrades to tropolone upon spontaneous decarboxylation. Further studies will be required to identify the enzymes for the final steps in 3,7-dHT biosynthesis, as no apparent candidates for these ring hydroxylation steps are encoded in the *Pseudomonas* BGCs.

### Comparative analysis of tropolone BGCs

Given the essential role of the majority of the *tpo* biosynthetic genes, we assessed the presence and diversity of this non-canonical class of BGCs across sequenced bacteria, especially as diverse tropolones are known to be produced across multiple genera and because these BGCs can be overlooked via automated genome mining methods. Due to the conserved requirement for TpoE-/TdaE-like flavoproteins across diverse tropolone biosynthetic pathways (39), we therefore used TpoE, TdaE^Pi,^ and TdaE^Bp^ as bait proteins to identify further tropolone BGCs.

**Fig 7:**
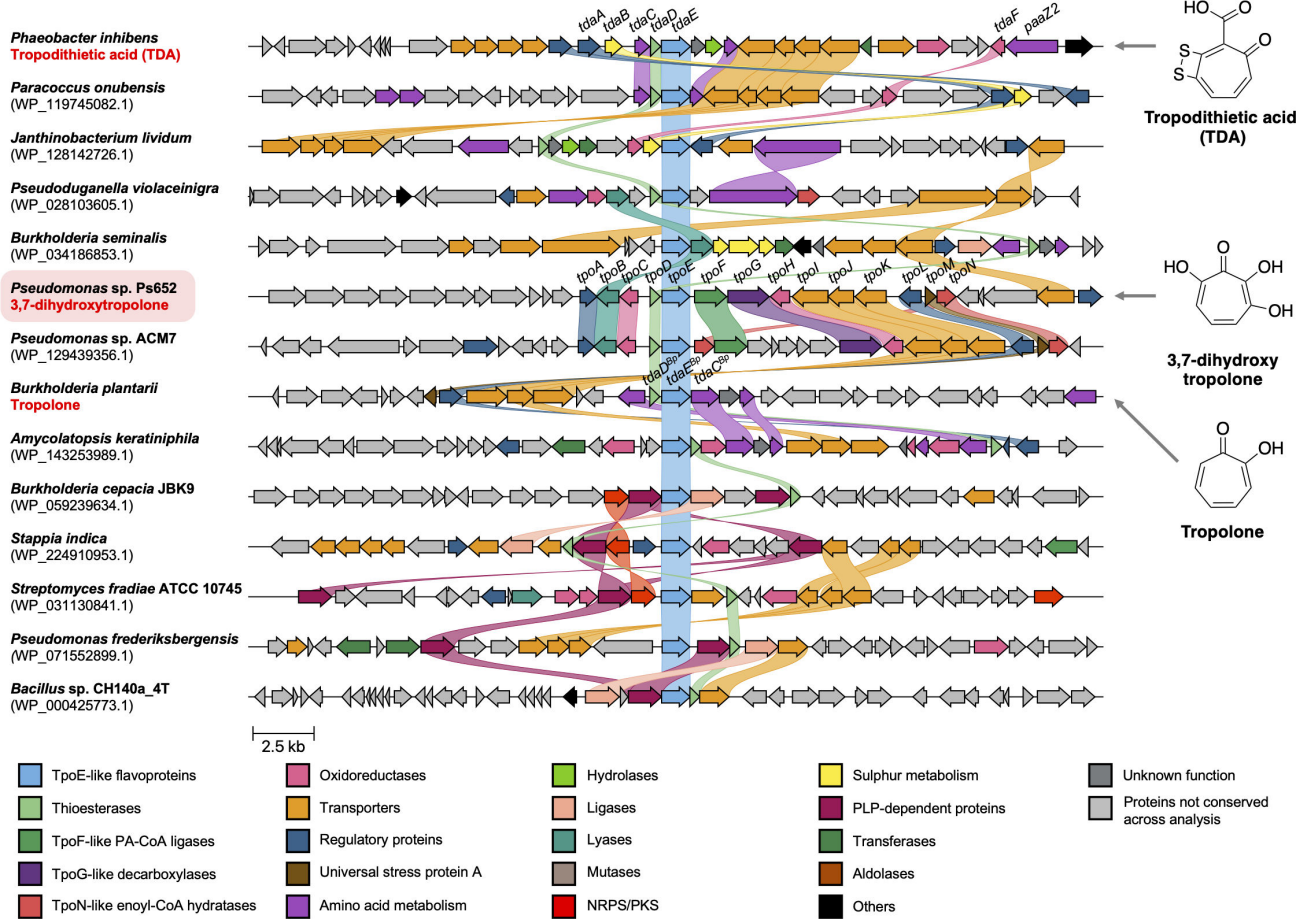
Comparison of bacterial tropolone BGCs and related BGCs identified by flavoprotein-led mining. BGC synteny is visualized using clinker (63), where genes with at least 30% identity are color-coded and linked. Previously characterized tropolone BGCs are highlighted, while the accessions of the TpoE homologs are listed for all other BGCs. Supplementary File 2 and Fig. S13 and S14 provide further BGC comparisons.

A strategy was devised to maximize the diversity of the resulting data set, where putative BGCs were retrieved as 35-kb regions centered on the *tpoE*/*tdaE* homologs (see Methods for full details). The resulting set of 319 BGCs were grouped into 67 BGC families using BiG-SCAPE (62) (Fig. S12). Diverse representatives of BGC families were then selected (86 clusters in total) for pan-family synteny analysis using clinker (63) (File S2; Fig. S13). A subset of these BGCs with tropolone-like features is shown in Fig. 7. An additional *Pseudomonas*-only analysis was performed to understand cluster distribution and synteny within this genus (Fig. S14). Mapping these BGCs to *Pseudomonas* phylogeny (Fig. S1) indicates that tropolone BGCs are rare but most commonly associated with the *Pseudomonas putida* group. The presence of the BGC in other *Pseudomonas* species groups supports some horizontal transfer within the genus.

We were interested in assessing whether the presence of PAA catabolon genes is a common feature of these BGCs, given the importance of TpoF, TpoG, and TpoN in 3,7-dHT biosynthesis (Fig. 4A and 5C). *tpoF* and *tpoG* homologs are always clustered in *Pseudomonas* BGCs, where a subset of BGCs feature additional PAA catabolon genes next to *tpoF*, such as *Pseudomonas* sp. ACM7, which has additional six PAA catabolon genes within the BGC (Fig. S14). In contrast, TpoF and TpoG homologs are not encoded in BGCs from other genera, which instead encode a variety of alternative proteins that potentially boost precursor supply and activate the PAA catabolon, such as PaaZ2 in *P. inhibens* and *Janthinobacterium lividum*, or 3-deoxy-7-phosphoheptulonate synthase and prephenate dehydratase in *Burkholderia*, *Trinickia,* and *Pseudoduganella*. This clustering of diverse PAA-associated genes with tropolone biosynthetic genes across varied BGCs suggests an uncommon form of convergent evolution that directs metabolic flux toward the biosynthesis of closely related specialized metabolites. Here, the tight regulation of the PAA catabolon could act as a selective pressure. Synteny across diverse BGCs also highlights potentially important roles for uncharacterized proteins, such as TpoB-like lyase/adolases (COG2321), which are conserved across BGCs in *Burkholderia*, *Serratia,* and *Pseudoduganella*, or transporter complexes with homologs across multiple BGCs.

Thioesterase TpoD has an essential role in 3,7-dHT biosynthesis. In contrast, previous *in vitro* enzyme assays suggested that a functional thioesterase may not be strictly required in *Burkholderia* and *P. inhibens* (39), even though the corresponding BGCs encode thioesterases (TdaD) with the same conserved domain (COG0824). Our genomic analysis demonstrated that TpoD-/TdaD-like thioesterases are encoded in all BGCs that are closely related to characterized tropolone BGCs. In addition, a different family of small thioesterase-like proteins (COG5496) are also associated with a subset of uncharacterized clusters from diverse Gram-negative and Gram-positive bacteria (such as *Amycolatopsis keratiniphila*, *Burkholderia cepacia,* and *Streptomyces fradiae*). These data hint at a conserved and important role for thioesterase-like proteins in tropolone biosynthesis, as well as providing lead BGCs for the discovery of novel troponoids (23, 24).

More broadly, the BiG-SCAPE and clinker data sets highlight the vast diversity of BGCs associated with TpoE-like flavoproteins. It is likely that many BGCs in the broader data set do not produce tropolones and are instead involved in alternative biosynthetic pathways, given the high frequency of diverse biosynthetic proteins encoded across clusters, including NRPSs, PKSs, transferases, amino acid metabolism proteins, ligases, aminotransferases, and oxidoreductases (Fig. 7; Fig. S13). This clustering indicates that TpoE/TdaE homologs are promising markers of uncharacterized BGCs involved in specialized metabolism, although experimental validation will be required to understand the true functions of these pathways.

## DISCUSSION

The tropolone chemophore is found in a variety of bioactive specialized metabolites (23, 24). These molecules have broad ecological relevance (64, 65) and medicinal promise, where some synthetic derivatives are under investigation as lead compounds for multiple diseases (66–69). Here, we have demonstrated that the tropolone (*tpo*) BGC in *Pseudomonas* sp. Ps652 is responsible for the biosynthesis of both 7-HT and 3,7-dHT, which represents the first report of a 3,7-dHT BGC in a Gram-negative bacterium. 3,7-dHT is produced in similar amounts to 7-HT (Fig. S4 and S8), which indicates that it could represent the true final product of the pathway, especially given that it is more active toward *S. scabies* (Fig. 4A and 7). In addition to the iron-binding activity of both molecules (18) (Fig. S7), the broad biological activity of both 7-HT and 3,7-dHT (69) indicates that both molecules could inhibit prokaryote and eukaryote organisms in competitive soil and plant microbial communities. Notably, the *tpo* BGC has been identified in multiple *Pseudomonas* strains via screens of large strain collections for organisms that inhibit plant pathogens (13, 21, 22, 70–72), highlighting the potential activity of these molecules among complex microbial communities. Future efforts should focus on understanding the function of tropolones in natural plant and soil colonization conditions, as well as the interplay with other BGCs and proteins encoded in tropolone producers (20, 73) (Fig. 2). For example, a recent work by Gram and colleagues has highlighted the impact of tropodithietic acid on *Phaeobacter* species and associated microbial communities (64, 65).

Based on mutational data and prior analyses of homologous BGCs in *Pseudomonas*, we propose a naming scheme for the *tpo* BGC (Fig. 3A). The high similarity of the Ps652 BGC to other characterized *Pseudomonas* suggests that these strains have the capacity to also produce 3,7-dHT, although this hypothesis needs to be tested. Using a mixture of metabolomics and reporter assays, we show that cluster-situated genes (*tpoFG*) generate PA-CoA, a key precursor for tropolone biosynthesis via the PAA catabolon, which is depressed by PA-CoA (Fig. 5A). This interplay between the PAA catabolon and tropolone biosynthesis is well-established in other organisms (23, 24), but the tight clustering of PAA genes within a tropolone BGC is unusual in Gram-negative bacteria. Informatic analysis indicates that some *Pseudomonas* strains have additional PAA genes embedded within tropolone BGCs (Fig. S14), which could further support metabolic flux toward tropolone biosynthesis. The involvement of the PAA catabolon in *Pseudomonas* biosynthesis of tropolones was highlighted in a recent study by Wang *et al*., which was conducted in parallel to our work (74), which showed that multiple PAA catabolon genes are essential for 7-HT production in *Pseudomonas donghuensis* HYS. The interplay between the PAA catabolon and 3,7-dHT biosynthesis also occurs in Actinobacteria (37, 38), where the 3,7-dHT biosynthetic pathway is different to the *Pseudomonas* pathway and therefore potentially represents convergent evolution toward a common strategy for tropolone production.

As in other tropolone pathways, our data are consistent with the proposal that PAA catabolism is intercepted via an enoyl-CoA dehydratase (TpoN), which generates a reactive intermediate that is converted into the 7-membered tropolone precursor (**3**, Fig. 5C). The previous cryptic role of an essential and conserved thioesterase (TpoD) could be elucidated in this work and involves the specific CoA-ester cleavage of **6** to afford **7** (Fig. 6), which spontaneously decomposes to tropolone. This is consistent with the results of a *lux* reporter assay (Fig. 5A) showing that the expression of the PAA catabolon is unaffected in the ∆*tpoD* mutant, which suggests that it functions downstream of the catabolon. The co-occurrence of thioesterases with TpoE-like flavoproteins in diverse BGCs (Fig. 7) is consistent with both enzymes acting in consecutive pathway steps, although biochemical assays showed that the dehydrogenase in *Burkholderia* (TdaE^Bp^) can catalyze CoA cleavage itself by an unusual O_2_-dependent mechanism (39). It will be interesting to see if TpoD homologs are essential in all tropolone biosynthetic pathways or whether TpoE homologs may in some cases suffice to ensure efficient CoA-ester cleavage. Further work is required to understand how the hydroxyl groups of 7-HT and 3,7-dHT are introduced. Characterized tropolone hydroxylases are flavoproteins (24), but there are no equivalent proteins in the *tpo* BGC. The BGC does encode two uncharacterized oxidoreductases (Fig. 3A) that may be somehow involved in these biosynthetic steps. Finally, we showed that TpoE/TdaE homologs can be used to identify uncharacterized BGCs via targeted genome mining, which are strong candidates for the production of novel metabolites, such as the ketoacyl synthase-associated BGCs (Fig. 7; Fig. S12 and S13).

## MATERIALS AND METHODS

### Chemicals and reagents

Unless otherwise specified, all chemicals were obtained from Sigma-Aldrich (Merck). All solvents for extractions and chromatographic applications were supplied by Fisher Scientific. Synthetic 7-HT was provided by Prof. Ryan Murelli (Brooklyn College, The City University of New York). All enzymes were obtained from New England Biolabs (NEB), unless other specified. DNA purification kits were obtained from Qiagen. Ultrapure water was obtained using a Milli-Q purification system from Merck. Oligonucleotides were obtained (desalted) from Sigma-Aldrich and redissolved in ultrapure Milli-Q water. All oligonucleotides used in this work are described in Table S2.

### Genome sequencing

A combined Illumina–Nanopore genome for *Pseudomonas* sp. Ps652 was obtained from MicrobesNG (Birmingham, UK). The strain was supplied for gDNA preparation as per MicrobesNG instructions. The supplied sequence was received as 5,972,118 bp in five contigs with 44.4 x coverage. The genome was reordered using the Align & Reorder contigs tool in Mauve (75) using *Pseudomonas* sp. SNU WT1 as a reference (76). The exported reordered file was submitted to the MeDuSa server (77) for scaffolding. The output scaffold produced was 5,972,218 bp in three contigs (5,966,768 bp, 5,323 bp, and 127 bp). Contig 2 (127 bp) and contig 3 (5,325 bp) were both found by BLAST (78) to be part of contig 1 (5,966,768 bp). These were thus removed, and 5,966,768 bp was used as the single scaffold genome, which was annotated using Prokka 1.13.3 (79) and submitted to the European Nucleotide Archive (Accession: OZ024668). The final genome has a size of 5.96 Mbp with 62.2% GC and 5,371 genes. This assembly was submitted to antiSMASH 7.0.0 (45) in July 2023 with “relaxed” detection strictness. The tropolone BGC has been deposited at MIBiG (80) with accession BGC0002843.

### Strains and media

All strains and plasmids used in this study are described in Tables S1 and S3. NEB 5-alpha competent *E. coli* cells (High Efficiency, C2987H, New England Biolabs) were used for all transformations of *E. coli* and performed as per the manufacturer’s instructions. All media used are defined in Table S4, where all formulae are for 1 L of deionized water, unless specified otherwise. All media were sterilized by autoclaving and standardized to pH 7.2, unless otherwise stated. All media ingredients were sourced from Sigma-Aldrich (Merck), unless otherwise stated. Antibiotics were added where necessary at the following concentrations: tetracycline (15 µg/mL for *E. coli*, 25 µg/mL for *Pseudomonas* sp. Ps652); gentamicin (25 µg/mL for *E. coli*, 50 µg/mL for *Pseudomonas*). *E. coli* was grown in liquid lysogeny broth (LB) medium at 30°C or 37°C or LB agar at 37°C for 16–20 hours. Stocks of *E. coli* strains were stored at −70°C in 25% glycerol. *Pseudomonas* strains were grown in Lennox medium at 30°C for 16–20 hours, unless otherwise specified. *Pseudomonas* strains were stored at −70°C as 925 µL overnight culture +75 µL DMSO or 500 µL overnight culture +500 µL 50% glycerol. *S. scabies* 87–22 were stored as spore stocks that were grown to sporulation on instant mash agar (IMA) plates, harvested in 20% glycerol, and stored at −70°C. *P. infestans* strain 88069 (The Sainsbury Laboratory, UK) was maintained on rye sucrose agar (RSA). Where relevant, all plates were imaged using a Canon Ixus 175 digital camera.

### Antimicrobial assays with *Streptomyces scabies* 87-22

Ten microliters of a *S. scabies* 87–22 spore stock was resuspended in 1 mL Milli-Q H_2_O, before 35 µL of this mixture was spread thoroughly onto IMA plates by using a sterile cotton bud. Fractions / extracts were dried *in vacuo* using a GeneVac and redissolved in 50–100 µL ethyl acetate and applied to filter paper disks, which were allowed to dry completely before being added to the IMA plate. Plates were incubated at 28°C for 2 days or until *S. scabies* had grown sufficiently for biological activity to be clearly observed.

### Assays with *P. infestans* strain 88069

A 5-mm diameter plug was taken from the actively growing outer edge of the *P. infestans* mycelium using a heat and ethanol sterilized number 3 metal corer and placed in the center of a standard Petri dish on RSA. Fifteen microliters of overnight liquid culture of the *Pseudomonas* strain to be tested was then spotted in duplicate 2.5 cm either side of the central plug. Spotted liquid culture was then dried in a biosafety level 2 cabinet before the plate was transferred to 20°C and incubated for 10 days.

### Iron binding assays

Iron binding was tested using chrome azurol S (CAS) agar plates as originally defined by Schwyn and Neilands (55). The binding of CAS / hexadecyltrimethylammonium (HDTMA) in the medium complexes with ferric iron to produce a blue color. Siderophores are able to compete for the binding of iron, liberating it from CAS/HDTMA, producing a color change from blue to orange. Five microliters of overnight culture or 5 µg of purified compounds dissolved in methanol (MeOH) were added as spots to the plate and allowed to dry. If overnight cultures were used, plates were incubated for 1 day at 30°C before being imaged. For pure compounds, the solvent was allowed to dry, and plates were then stored at room temperature overnight to allow for diffusion of applied molecules before being imaged.

### In-frame deletions of *Pseudomonas* genes

In-frame deletion mutants of *Pseudomonas* strains were constructed by markerless two-step allelic exchange (81). Mutant alleles were designed by selecting approximately 500-bp sequences upstream and downstream of the region to be deleted, in-frame with the gene(s) to be deleted. These were either amplified by the polymerase chain reaction (PCR) (primers in Table S2) and cloned into the pTS1 suicide vector (8) by Gibson assembly, or synthesized by Twist Biosciences Ltd., where the genes were provided in the pTS1 vector. Gibson assembly was performed using Gibson Assembly Master Mix or NEBuilder HiFi DNA Assembly mix according to the manufacturer’s instructions. The pTS1 plasmid containing the shortened, mutant allele was used to transform *Pseudomonas* sp. Ps652 as follows. Ps652 was streaked from glycerol stocks onto Lennox agar and grown at 30°C until visible colonies appeared. Single colonies were used to inoculate 10 mL Lennox broth per transformation. Liquid cultures were grown overnight before being centrifuged at 4,000 x g for 8 minutes and resuspended in sterile 300 mM sucrose (2 mL). 2 × 1 mL was transferred to two 2-mL microcentrifuge tubes and centrifuged at 11,000 x g for 1 minute, and the supernatant was removed by decanting. One milliliter 300 mM sucrose was added and resuspended by gentle vortex mixing. This was centrifuged at 11,000 x g for 1 minute, and the supernatant was removed as before. This wash was repeated twice more, and the cells from both tubes were resuspended in a total of 100 µL 300 mM sucrose. Two microliters of DNA was added to each 100-µL aliquot and mixed carefully with a pipette tip.

Samples were then added to electroporation cuvettes (2 mm), and electroporation was performed using an Eppendorf Eporator at a setting of 2,500 V. Lennox broth (900 µL) was added immediately after electroporation, and the samples were mixed by gentle pipetting. Electroporated cells were incubated at 30°C for 1 hour with shaking at 250 rpm. One hundred microliters of electroporated cells was then plated on Lennox agar with antibiotics as required and incubated at 28°C until colonies were visible. Individual colonies were inoculated into 10 mL Lennox broth and grown for 16–18 hours at 28°C with 250 rpm shaking. These cultures were then diluted to 10^−6^ and plated on Lennox agar supplemented with 10% sucrose and incubated at 28°C until visible colonies were formed. These colonies were restreaked and then checked by colony PCR with GoTaq 2X Master Mix for the mutant allele, with the parent strain as the control. Positive clones were restreaked again to single colonies, tested again, and then grown for 16–18 hours at 28°C with 250 rpm shaking in Lennox broth supplemented with tetracycline. DMSO (75 µL) was added to 925 µL of the culture, and this was stored at −70°C.

### Preparation of extracts from *tpo* mutants

All strains were streaked from stocks to single colonies on Lennox agar plates with selection if necessary and grown overnight in biological triplicate in 5 mL MKBG in 50-mL centrifuge tubes with sterilized foam bungs and no selection to prevent background activity from added antibiotics. Five milliliters of EtOAc was added, and tubes were agitated for 1 hour at 250 rpm. These were then centrifuged at 4,000 x g for 5 minutes to pellet any cell debris and ensure proper separation of aqueous and organic phases. 1.5-mL samples of the organic phase were taken for each and dried fully by centrifugation under reduced pressure at 30°C on a Genevac EZ-2 Plus system using the “Low BP” setting, before being redissolved in 1.5 mL 100% MeOH and stored at −30°C until use.

### Complementation of mutants

Ps652 ∆*hcn*∆*tpoD* was complemented by PCR amplification of *tpoD* and the entire intergenic region preceding it (primers in Table S2) and cloning into the multicopy replicative vector pME6032 (53) by Gibson assembly using Gibson Assembly Master Mix or NEBuilder HiFi DNA Assembly mix according to the manufacturer’s instructions. Assembled constructs were transformed into NEB 5-alpha *E. coli* according to the manufacturer’s instructions and described above and plated on LB agar supplemented with 15 µg/mL tetracycline. Plasmids were isolated from replicate lines, and clones positive for pME6032-*tpoD* were identified by the PCR with the prepared plasmid as the template (primers in Table S2). Ps652 ∆*hcn*∆*tpoD* was transformed with pME6032 or pME6032-*tpoD* by electroporation as described above for in-frame deletion mutants and plated on Lennox agar supplemented with 25 µg/mL tetracycline. Single colonies were inoculated into 10 mL Lennox broth and plasmids isolated, followed by diagnostic digest with BamHI and HindIII, revealing an insert of the expected size. Positive clones were maintained as described above.

Both *tpoE* and *tpoF* were complemented using the pUC18-mini-Tn7-Gm gentamicin-selective vector (Table S3). In each case, the 5′-UTR of *tpoD* was PCR-amplified along with *tpoE* / *tpoF* and cloned into the BamHI site of pUC18-mini-Tn7-Gm by Gibson assembly, with the *tpoD* 5′-UTR directly upstream of *tpoE* / *tpoF* to allow native regulation. Assembled constructs were transformed into NEB 5-alpha *E. coli* cells as described above with selection on 25 µg/mL gentamicin. Plasmids were isolated from replicate lines and screened by PCR amplification of the whole insert (5′-UTR and *tpoE* / *tpoF*). Positive clones were maintained as described above. Ps652 ∆*hcn*∆*tpoE* and Ps652 ∆*hcn*∆*tpoF* were transformed with the corresponding constructs, along with the pTNS2 helper plasmid (56), by electroporation as described above, and selected on Lennox agar supplemented with 50 µg/mL gentamicin. Clones carrying the insertion were identified by PCR for the whole insert (primers in Table S2).

Chemical complementation was performed for Ps652 ∆*hcn*∆*tpoE* and Ps652 ∆*hcn*∆*tpoF* by preparing extracts as described above with three replicates per strain, with the addition of 1 mM phenylacetic acid (PAA) as appropriate to the MKBG medium.

### Liquid chromatography–mass spectrometry (LC-MS)

LC-MS/MS analysis was performed on a Q-Exactive mass spectrometry system (Thermo Fisher Scientific). A Luna Omega 1.6 µm Polar C18 100 Å (50 × 2.1 mm) column (Phenomenex) was used with 0.1% formic acid in Milli-Q H_2_O and MeOH as the mobile phase. Gradients were linear 0%–95% MeOH from 1 to 6 minutes, followed by 95% MeOH until 8.8 minutes to wash off any material still associated with the column. Spectra were acquired in either the positive or negative mode using a scan range of *m/z* 50–500. Mass spectra were obtained in the positive mode using full MS/dd-MS^2^ acquisition settings with the following specific parameters: chromatography peak width = 7 s; full MS settings: resolution = 70,000, AGC target = 3×10^6^, maximum IT = 100 ms, scan range 50 to 500 *m/z*; dd-MS^2^ settings: resolution = 17,500, AGC target = 1×10^5^, maximum IT = 50 ms, loop count = 5, isolation window 1.5 *m/z*, (N)CE/ stepped nce 20, 40, 60; dd settings: minimum AGC target = 8×10^3^, exclude isotopes ON, dynamic exclusion = 1 second. Additionally for some runs, single ion monitoring (SIM) was performed in parallel with the data acquisition previously described using the following parameters: resolution = 70,000, AGC target = 5×10^4^, maximum IT = 200 ms, scan range 150 to 2,000 *m/z*, isolation window 1.5 *m/z,* inclusion masses *m/z* 139.10140 (M + H, positive) and *m/z* 155.03360 (M + H, positive).

### Production and extraction of 3,7-dihydroxytropolone

To avoid reisolating pyoverdine when using UV absorption to track tropolones throughout purification, a pyoverdine-null mutant (Ps652 *∆hcn∆pyo*) was used for production and purification of tropolones. Ps652 *∆hcn∆pyo* was grown for 16 hours in 2 × 10 mL MKBG in plastic universal 30-mL tubes. This culture was then used to inoculate 2 L of MKBG in 200 × 10 mL aliquots in 50-mL centrifuge tubes with foam bungs, using 100 µL seed culture per 10 mL, and then grown for 24 hours at 30°C with shaking at 250 rpm. The culture from all 200 tubes was then combined into a single 2 L volume. Cells were removed by centrifugation at 8,000 x g for 8 minutes at 4°C in a Sorvall Lynx 6000 centrifuge (Thermo Fisher Scientific). The supernatant was collected and cells discarded. The supernatant was concentrated by rotary evaporation to a volume of 1 L. Following the method of Jiang *et al*. (18), the supernatant was then extracted three times with 0.5 L of EtOAc before being acidified to pH 2 using HCl. The aqueous phase was then extracted three more times with 0.5 L to yield a total of 3 liters of the organic extract. All organic extracts were combined and dried by rotary evaporation.

### Purification of 3,7-dihydroxytropolone

The dried organic extract was dissolved in 3 mL MeOH and subjected to size-exclusion chromatography using a Sephadex LH-20 column on an ÄKTA Pure system (Cytiva) in 100% MeOH attached to an Optilab differential refractive index detector (Wyatt Technology). The flow rate was 1 mL/min, and 10-mL fractions were collected from 350 minutes to 700 minutes. UV absorbance was monitored at 330 nm to detect tropolones. The 3 mL of the organic extract was processed in two injections. All fractions were dried on a Genevac EZ-2 Plus system using the “Low BP” setting at 30°C, and all fractions with strong absorbance at 330 nm were redissolved in MeOH, diluted 1:5, and 5 µL analyzed by LC-MS on a Q-Exactive Orbitrap mass spectrometer (Thermo Fisher). Several fractions showed high levels of 7-hydroxytropolone, 3,7-dihydroxytropolone, or a mixture of both. These fractions were selected for further purification.

Fractions containing hydroxylated tropolones were redissolved in 100% MeOH, and fractions with similar LC-MS profiles were combined. These fractions were processed by semi-preparative HPLC on a Dionex Ultimate 3000 system (Thermo Fisher Scientific) with a Luna Omega 5 µm Polar C18 100 Å 250 × 10 mm column (Phenomenex) in five injections. A multistep gradient of Milli-Q H_2_O + 0.5% formic acid and MeOH +0.5% formic acid was used (see Table S7 for details) with a flow rate of 4 mL/min, and UV absorbance was monitored at 254 nm and 327 nm. The chromatogram showed two clear peaks at 327 nm, with the earlier peak representing 3,7-dihydroxytropolone and the later peak representing the 7-hydroxytropolone (Fig. S4). All fractions corresponding to 3,7-dihydroxytropolone were combined and dried overnight on a Genevac EZ-2 Plus system using the “HPLC Lyo” setting at 30°C. 3,7-dihydroxytropolone (2.8 mg) was obtained and was observed to be a white powder with a slight pink hue.

### NMR analysis of 3,7-dihydroxytropolone

3,7-dihydroxytropolone (2.8 mg) was redissolved in 500 µL CD_3_OD and shaken for 10 minutes to exchange OH protons for deuterium and dried on a Genevac EZ-2 Plus system on the “Low BP” setting at 30°C. The sample was then again redissolved in 500 µL CD_3_OD and analyzed on a Bruker AVANCE NEO 600 MHz spectrometer equipped with a TCI cryoprobe. The experiments were carried out at 298 K with the residual CD_3_OD solvent used as an internal standard (δ_H_/δ_C_ 3.31/49.0 ppm). Resonances were assigned through 1D ^1^H and ^13^C experiments. Spectra were analyzed using Bruker TopSpin 3.5 software. NMR spectra are reported in Fig. S5 and S6. ^1^H NMR ∂= 6.98 (3H, m). ^13^C NMR ∂= 119.24 (2C), 129.44, 158.11 (2C), and 158.94 (2C).

### Reporter assay for PAA catabolon

The 5′-UTR of *paaA* was PCR-amplified (primers in Table S2) and cloned by Gibson assembly into the BamHI to the XcmI site of pUC18-mini-Tn7-Gm-lux plasmid upstream of *luxC*. Assembled constructs were transformed into NEB 5-alpha *E. coli* as described above and plated on LB agar supplemented with 25 µg/mL gentamicin. Plasmids were isolated from replicate lines, amplified by PCR (primers in Table S2), and verified by Sanger sequencing (Eurofins). In order to optimize the distance between the ribosome-binding site and the start of *luxC*, site-directed mutagenesis via PCR (Table S2) was performed using Phire Hot Start II DNA Polymerase (Thermo Fisher Scientific) and again verified by Sanger sequencing. pUC18-mini-Tn7-Gm-paa-lux was transformed, along with the pTNS2 helper plasmid, into the relevant Ps652 strains by electroporation, as described above, and selected on gentamicin. Positive clones were identified by PCR screening (primers in Table S2).

For luminescence measurements, all strains were inoculated from single colonies into 10 mL MKBG medium with or without addition of 1 mM PAA and grown for 12 hours at 30°C with shaking at 250 rpm. All cultures were then diluted to an optical density at 600 nm of 1.0 in the relevant medium in 1.5-mL microcentrifuge tubes per strain (five replicates) and shaken for 1 hour at 30°C. Luminescence measurements were then taken on a GloMax-Multi Jr instrument (Promega).

### Analysis of flavoprotein-associated BGCs

The flavoproteins from *Pseudomonas* sp. Ps652 (TpoE, VVM49077.1), *Burkholderia plantarii* (TdaE^Bp^, WP_042624079.1), and *Phaeobacter inhibens* (TdaE^Pi^, WP_014881725.1) were used as queries for BlastP searches using the NCBI non-redundant protein database with default search paraments (78). The top 1,000 hits were retrieved from each search (3,000 accessions in total), which were then filtered for duplicates to 1,543 accessions. This list of proteins was then filtered using a 95% sequence identity cut-off using CD-Hit (82) to reduce redundancy in downstream analyses to provide a list of 526 protein accessions whose identity to each other was lower than 95%. This accession list was then used to retrieve 35-kb Genbank files centred on the input accessions. The data set was further filtered to remove poor-quality output files that resulted from poor sequencing data, such as from metagenome-assembled genomes and contig edges of isolated genomes. Additional output files from non-bacterial sources were also removed. The final output consisted of 319 putative BGCs as Genbank files (Supplementary File 1).

The Genbank files were each edited to make them compatible with features within BiG-SCAPE (62) 1.1.5 (glocal mode and anchor domains) by adding a /product="other" qualifier following the region feature and a /gene_kind="biosynthetic" qualifier to the CDS entry for the TdaE/TdoE homolog in each file. BiG-SCAPE 1.1.5 was run using the following parameters with the 319 BGCs plus the tropodithietic acid BGC (MiBIG BGC0000932):

--mix --mode glocal—cutoffs 0.5 0.75 0.85—anchorfile anchor_domains.txt—include_singletons --clans-off

The anchor file included the following information to anchor the BGC analysis on the central flavoprotein of each cluster:

PF00441 Dehydrogenase_C-term [Others]

PF02771 Dehydrogenase_N-term [Others]

Manual assessment determined that a cut-off of 0.75 separated clusters into meaningful families without overly fragmenting the output into a large proportion of singletons. In total, the clusters were grouped into 67 families, which were further visualized and annotated using Cytoscape 3.10.1 (Fig. S12). Relatively permissive networking parameters were used due to the overall diversity of the data set and the lack of multiple “anchor domains” that BiG-SCAPE usually uses for well-characterized classes of BGC.

To visualize the synteny and diversity of the resulting families, one or two representative clusters were selected from each family and visualized using clinker (63) 0.0.27 (Fig. 7; Fig. S13; Supplementary File 2).

### Analysis of *Pseudomonas* phylogeny and tropolone BGC synteny

TpoE (VVM49077.1) was used as a query for BlastP searches using only *Pseudomonas* sequences within the NCBI non-redundant protein database and with a 50% identity cut-off. A total of 22 proteins were retrieved, which were then filtered using a 99% sequence identity cut-off using CD-Hit (82) to provide eight protein accessions. 35-kb Genbank files were obtained that were centered on each protein accession. BGC synteny was then visualized using clinker (63) 0.0.27 (Fig. S14). To assess the taxonomy of Ps652 and the associated distribution of tropolone BGCs, the genomes of Ps652 and the seven additional strains were used as input for AutoMLST (40). Default AutoMLST settings were used plus ModelFinder and IQ-Tree bootstrapping. AutoMLST automatically picks reference strains (up to a maximum of 50 strains for the tree) for the remainder of the tree and associated conserved genes for multilocus phylogenetic analysis. The precise strains used for the tree were manually adjusted to reduce taxonomic redundancy and obtain a broader representation of the *Pseudomonas* genus. The conserved genes selected by AutoMLST for tree building are listed in Supplementary File 1. In addition to the eight input strains, the other strains in the tree were assessed for the presence of a tropolone BGC. The resulting tree was visualized using iTOL (83) (Fig. S1).

### Cloning and recombinant protein production of TpoE and TpoD

The genes *tpoE* and *tpoD* were synthesized and codon-optimized for *E. coli* by Biocat (Heidelberg, Germany). Using restriction enzyme digestion with NotI (3’) and NcoI (5’), both fragments were cloned in a modified pET28b-vector, which adds an N-terminal His6x-gb1-Tag to the recombinant protein. This vector contained a hexahistidine-tag for immobilized metal-affinity chromatography (IMAC) purification, a solubility enhancer gb1-tag (B1 domain of *Streptococcal* protein g), and a TEV protease cleavage site. Correct cloning was confirmed by Sanger sequencing.

The resulting constructs were transformed in *E. coli* BL21 (DE3) pL1SL2 cells (84). These cells contain a GroES/GroEL chaperonin system, which helps with protein folding. Single colonies were picked, and starter cultures were grown in LB medium supplemented with 50 µg/mL kanamycin, 100 µg/mL ampicillin, and 20 µg/mL chloramphenicol at 37°C and 130 rpm overnight. Main cultures with the TB medium and the same amount of antibiotics were inoculated with 1% of the overnight cultures and grown at 37°C and 130 rpm to an OD_600nm_ between 0.4 and 0.6. After that, protein expression was induced with 500 µM IPTG. The cultures were subsequently incubated at 18°C and 130 rpm until the following day. The cultures were harvested by centrifugation at 5,000 x g for 30 minutes at 4°C, resuspended in 0.9% NaCl (to remove the residual medium), centrifuged again with the same parameters, and subsequently frozen as pellet at −20°C.

### Protein purification of TpoE and TpoD

The frozen cell pellets were resuspended in lysis buffer containing 300 mM NaCl, 10% glycerol (vol/vol), 50 mM sodium phosphate buffer (Na_2_HPO_4_/NaH_2_PO_4_) at pH 7.4. The resuspended cells were lysed by ultrasonication (3 s pulse, 3 s pause, 4 min pulse time, amplitude: 60%, repeated once). The lysate was cleared by centrifugation (18,000 x g, 30 minutes and 4°C) and sterile filtration (pore size 0.22 µm) and applied to an Ni-NTA column (Cytiva). The column was washed with lysis buffer containing an additional 30 mM imidazole to remove other protein impurities. Elution of the target protein was then carried out with lysis buffer supplemented with 500 mM imidazole. Subsequently, a desalting column was used to exchange buffer and remove imidazole. The obtained fractions were concentrated with an Amicon concentrator (Merck Millipore) and flash-frozen in liquid nitrogen upon storage at −80°C.

### Recombinant protein production and purification of PaaABCE, PaaG, and PaaZ-E256Q

Enzymes from the PAA catabolon for the *in vitro* formation of tropolone precursor **3** were produced and purified as previously described (30, 32, 34).

### Enzymatic synthesis of 7

Compound **7**, the precursor for tropolone formation, was synthesized using the same substrate, enzymes, and reaction conditions as previously described (39).

### Turnover assay with TpoE and TpoD

To the substrate mix containing approximately 0.5 mM **3** (enzymatically produced by PaaABCE, PaaG & PaaZ-E256Q) in Tris-HCl 50 mM buffer either 1 µM of TpoE, 1 µM of TpoD, or 1 µM of both enzymes was added. The samples were put on a tabletop shaker in Eppendorf tubes with open lids and incubated for 15 minutes at 30°C and vigorous shaking (900 rpm). Afterward, the reaction was quenched by adding an equal volume of ethyl acetate with 0.1% formic acid and thorough vortexing of the samples. The samples were centrifuged at 18,000 x g for 10 minutes, and the organic phase was separated from the aqueous phase. The organic ethyl acetate phase was dried under N_2_ gas flow, and the samples were resuspended in acetonitrile.

### RP-HPLC-MS analysis of the enzyme assays

Organic and aqueous phases obtained from TpoE/TpoD assays were both analyzed by RP-HPLC on a Shimadzu LCMS-8030 Triple Quad Mass Spectrometer. A Sunfire C18 column (150 × 3 mm ID, 3.5 µM, Waters) with a guard column was used. For aqueous samples, 10 mM ammonium acetate buffer pH 4.5 was used as solution A1, for organic samples water +0.1% formic acid (A2) was used. Solution B1 was acetonitrile, and B2 was acetonitrile +0.1% formic acid. The HPLC program for both aqueous and organic phases was the same. The flow rate was set to 0.4 mL/minute, and the following gradient was used:

2%–12% B (0–4 minutes), 12% B (4–6 minutes), 12%–60% B (6–14 minutes), 60% B (14–18 minutes), 60%–100% B (18–19 minutes), 100% B (19–22 minutes), 100%–2% B (22–23 minutes), 2% B (23–26 minutes).

Absorption was measured from 190 to 800 nm. For MS measurements, electrospray ionization (ESI) was used in both negative and positive modes with the following settings: 3 kV capillary voltage, 400°C heat block temperature, 250°C DL temperature, and 3 L/min nebulizing gas flow.

## Data Availability

The Ps652 genome has been deposited at the European Nucleotide Archive (Accession: OZ024668). BGC data will be available at MIBiG (80) with accession BGC0002843. LC-MS data are deposited as a MassIVE data set (MSV000095461). All other data generated in this study are included in this manuscript and its supplementary ﬁles.

## SUPPLEMENTAL MATERIAL

File S1 mbio.01022-24-s0001.xlsx Information associated with lux assays, genome mining, and phylogenetic analysis.

File S2 mbio.01022-24-s0002.pdf Expanded biosynthetic gene cluster synteny visualization.

Supplemental Information mbio.01022-24-s0003.pdf Supplemental tables and figures.

ASM does not own the copyrights to Supplemental Material that may be linked to, or accessed through, an article. The authors have granted ASM a non-exclusive, world-wide license to publish the Supplemental Material files. Please contact the corresponding author directly for reuse.
