# Supplementary material for: Understanding the biosynthesis, metabolic regulation, and anti-phytopathogen activity of 3,7-dihydroxytropolone in Pseudomonas spp
Source: mBio. 2024 Aug 29;15(10):e01022-24. doi: 10.1128/mbio.01022-24 (PMC11481866; doi:10.1128/mbio.01022-24)
Supplement: File S2 — Expanded biosynthetic gene cluster synteny visualization. [file mbio.01022-24-s0002.pdf]

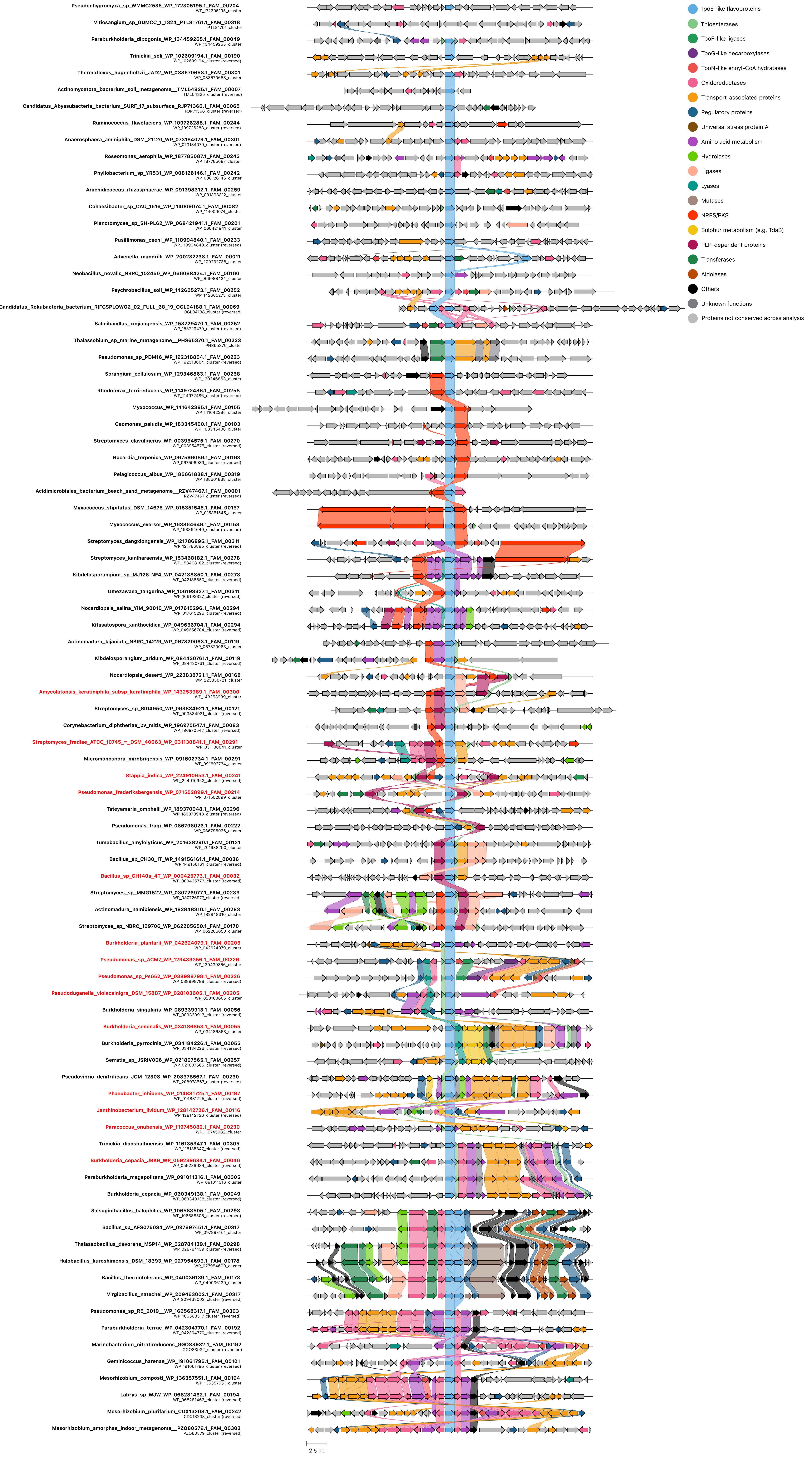

Supplementary File 2 Comparison of biosynthetic gene clusters (BGCs) that are representative of families identified using a flavoprotein-led genome mining approach. Synteny is visualised using clinker (Gilchrist CLM, Chooi Y-H. 2021 Bioinformatics 37:2473–2475), where genes with at least 30% identity are colour-coded and linked. The accessions of the TpoE homologues are listed for all BGCs, along with the BGC family number determined via BiG-SCAPE (Navarro-Muñoz JC, et al. 2020 Nat Chem Biol 16:60–68) analysis. BGCs shown in Figure 7 are highlighted by red text.
