# Supplementary material for: Understanding the biosynthesis, metabolic regulation, and anti-phytopathogen activity of 3,7-dihydroxytropolone in Pseudomonas spp
Source: mBio. 2024 Aug 29;15(10):e01022-24. doi: 10.1128/mbio.01022-24 (PMC11481866; doi:10.1128/mbio.01022-24)
Supplement: Supplemental Information — Supplemental tables and figures. [file mbio.01022-24-s0003.pdf]

SUPPORTING INFORMATION

Understanding the biosynthesis, metabolic regulation, and anti-phytopathogen activity of 3,7-dihydroxytropolone in *Pseudomonas* spp.

Alaster D. Moffat[1], Lars Höing[2], Javier Santos-Aberturas[1], Tim Markwalder[2], Jacob G. Malone[1,3], Robin Teufel[2] and Andrew W. Truman[1]*

1. Department of Molecular Microbiology, John Innes Centre, Norwich, NR4 7UH, United Kingdom.
2. Department of Pharmaceutical Sciences, University of Basel, 4056, Basel, Switzerland.
3. School of Biological Sciences, University of East Anglia, Norwich, NR4 7TU, United Kingdom.

CONTENTS

Table S1 Strains used in this study 2
Table S2 Oligonucleotides used in this study 3
Table S3 Plasmids used in this study 5
Table S4 Media used in this study 6
Table S5 Biosynthetic gene clusters in Ps652 7
Table S6 Ps652 tropolone biosynthetic gene cluster 8
Table S7 Gradient for semi-preparative HPLC 9
Figure S1 Phylogenetic analysis of Ps652 versus other *Pseudomonas* strains 10
Figure S2 Volatile activity versus *P. infestans* using different media 11
Figure S3 Detection of tropolones in Ps652. 11
Figure S4 Separation of tropolones using HPLC 12
Figure S5 ^{1}H NMR spectrum of 3,7-dHT 13
Figure S6 ^{13}C NMR spectrum of 3,7-dHT 14
Figure S7 Antibacterial and iron binding activities of 7-HT and 3,7-dHT 15
Figure S8 LC-MS analysis of complementations for Δ*tpoE* and Δ*tpoF* 16
Figure S9 PAA catabolon in Ps652 17
Figure S10 LC-MS analysis of TpoD and TpoE reactions 18
Figure S11 Comparison of TpoD/TpoE and $TdaE^{Pi}$ reactions 18
Figure S12 BGC networking from genome mining 19
Figure S13 Example BGCs featuring PKSs or NRPSs 20
Figure S14 Comparison of *Pseudomonas* BGCs. 21
References 22

TABLES

Table S1 Strains used in this study.

Strain	Genotype / description	Application	Reference
Ps652	*Pseudomonas* environmental isolate	Environmental biocontrol isolate	1
Ps652 Δ*hcn*	HCN null mutant (Δ*hcnABC*)	Investigation of Ps652 bioactivity	This work
Ps652 Δ*hcn*Δ*pyo*	Pyoverdine null mutant	Investigation of Ps652 bioactivity	This work
Ps652ΔNRPS5	NRPS5 null mutant	Investigation of Ps652 bioactivity	This work
Ps652 Δ*hcn* 29C1	Ps652 Δ*hcn* with transposon insertion mutant *tpoJ*	Investigation of *tpo* gene cluster	This work
Ps652 Δ*hcn*Δ*tpoD*	Δ*hcnABC*Δ*tpoD*	Investigation of function of thioesterase TpoD	This work
Ps652 Δ*hcn*Δ*tpoE*	Δ*hcnABC*Δ*tpoE*	Investigation of function of dehydrogenase TpoE	This work
Ps652 Δ*hcn*Δ*tpoF*	Δ*hcnABC*Δ*tpoF*	Investigation of function of acyl-CoA ligase TpoF	This work
Ps652 Δ*hcn*Δ*tpoG*	Δ*hcnABC*Δ*tpoG*	Investigation of function of decarboxylase TpoG	This work
Ps652 Δ*hcn*Δ*tpoN*	Δ*hcnABC*Δ*tpoN*	Investigation of function of enoyl-CoA hydratase TpoN	This work
Ps652 ΔhcnΔ*tpoD*:: pME6032	Empty vector control for *tpoD* complementation	Investigation of function of thioesterase TpoD	This work
Ps652 ΔhcnΔ*tpoD*:: pME6032-*tpoD*	Δ*hcnABC*Δ*tpoD* complemented with *tpoD* in multicopy expression vector pME6032	Investigation of function of thioesterase TpoD	This work
Escherichia coli NEB 5-alpha (Subcloning Efficiency)	*fhuA2 Δ(argF-lacZ)U169 phoA glnV44 Φ80 Δ(lacZ)M15 gyrA96 recA1 relA1 endA1 thi-1 hsdR17*	Plasmid host for molecular cloning	New England Biolabs[2]
Escherichia coli BL21 (DE3) pL1SL2	*E. coli* BL21 (DE3) with GroES/GroEL chaperonin system	Protein expression with chaperonins to help protein folding	3
Streptomyces scabies 87-22	Wild type	Bacterial potato pathogen for assays	4
Phytophthora infestans strain 88069	Wild type	Oomycete potato pathogen for assays	5

Table S2 Oligonucleotides used in this study.

Primer	Sequence 5'-3'	Application
HCN_UP_F	CAGAGAGGAGCACATTCACG	Amplification of Upstream HCN fragment (forward)
HCN_UP_R	CAGTTGATCATGGTCGTTGC	Amplification of upstream HCN fragment (reverse)
HCN_DOWN_F	CCTTCCACTGCGATACCCTG	Amplification of downstream HCN fragment (forward)
HCN_DOWN_R	CCTGCTTGTTTTCCACAGCC	Amplification of downstream HCN fragment (reverse)
tpoD_del_fwd	ACCGAGAGCAGCAGTTCC	PCR screening of Ps652 Δ*tpoD* mutant
tpoD_del_rev	ATCAACAGCACTCCTTGAGG	PCR screening of Ps652 Δ*tpoD* mutant
tpoD_del_seq	GACCACTTCCTTGCTGTCG	Sequencing of Ps652 Δ*tpoD* mutants
tpoF_del_fwd	CGTAGGTGGTCAGTACC	PCR screening of Ps652 Δ*tpoF* mutant
tpoF_del_rev	AGGATGCATTGGGCTTCG	PCR screening of Ps652 Δ*tpoF* mutant
NRPS_del_fwd	GAACTCGCGATCACTAACC	PCR screening of Ps652 NRPS (BGC6) mutants
NRPS_del_rev	CTGGAATCGGATCGAAGTCG	PCR screening of Ps652 NRPS (BGC6) mutants
tpoN_del_fwd	CTTACCAGTTTCTGCAGG	PCR screening of Ps652 Δ*tpoN* mutant
tpoN_del_rev	GGTGCGATTCATATCG	PCR screening of Ps652 Δ*tpoN* mutant
tpoD_comp_ PME6032_F	AAGCCGCCACGGCGATATCGGATCCAT CAACAGCACTCCTTGAGG	Cloning *tpoD* into pME6032
tpoD_comp_ PME6032_R	CCATGGTACCCGGGAGCTCGAATTCTC AGCCTTCCTGGAACG	Cloning *tpoD* into pME6032
tpoG_UP_Fwd	GCTCGGTACCCGGGGATCCTCTAGAAT GGCCGAGCGTGAATGG	Deletion of *tpoG* gene in Ps652 Δ*hcn*
tpoG_UP_Rev	GGAATTCGGCATCCGCCATCTGTTTGC CGAGTTCGACTACG	Deletion of *tpoG* gene in Ps652 Δ*hcn*
tpoG_DOWN_Fwd	ATGGCGGATGCCGAATTCC	Deletion of *tpoG* gene in Ps652 Δ*hcn*
tpoG_DOWN_Rev	GGTGAGAATGGCAAAAGCTCATATGGT CCTGACCACCCTGAGC	Deletion of *tpoG* gene in Ps652 Δ*hcn*
tpoG_del_Fwd_2	GACATCAGCGACGATGACTGG	PCR screening of Ps652 Δ*tpoG* mutants
tpoG_del_Rev	TGAGCAAGGAAGCACTGC	PCR screening of Ps652 Δ*tpoG* mutants
tpoE_del_Fwd	GTTCACGATGAGCAACAAGG	PCR screening of Ps652 Δ*tpoE* mutants
tpoE_del_Rev	GACCACGGTCGGTTTGC	PCR screening of Ps652 Δ*tpoE* mutants

Primer	Sequence 5'-3'	Application
tpo_comp_UP_F	TCATGCATGAGCTCACTAGTGGATCATC AACAGCACTCCTTGAGG	Amplification of *tpoD* promoter for cloning *tpo* genes into pUC18-mini-Tn7-Gm; PCR screening of complemented mutants
tpoE_comp_UP_R	GGAGTCCAGGAGAGTTTCATCGTGAAC GTCCTTTTATTGATTTGTGG	Amplification of tpoD promoter for cloning *tpoE* into pUC18-mini-Tn7-Gm
tpoE_comp_DOWN_F	ATGAAACTCTCCTGGACTCC	Cloning *tpoE* into pUC18-mini-Tn7-Gm
tpoE_comp_DOWN_R	AGGAATTCCTGCAGCCCGGGGGATCTG GCTCGACCGCTTCAGG	Cloning *tpoE* into pUC18-mini-Tn7-Gm; PCR screening of complemented mutants
tpoF_comp_UP_R	GGTATCGATTTGGTTGGCATCGTGAAC GTCCTTTTATTGATTTGTGG	Amplification of tpoD promoter for cloning *tpoF* into pUC18-mini-Tn7-Gm
tpoF_comp_DOWN_F	ATGCCAACCAAATCGATACC	Cloning *tpoF* into pUC18-mini-Tn7-Gm
tpoF_comp_DOWN_R	AGGAATTCCTGCAGCCCGGGGGATCCT CGTGAGTCCTTTGAGG	Cloning *tpoF* into pUC18-mini-Tn7-Gm; PCR screening of complemented mutants
PAA_prom_fwd	CATGCATGAGCTCACTAGTGGATCCTTA GGTTCTTCAGGCCATCC	Cloning *paaA* 5'-UTR into pUC18-mini-Tn7-Gm-lux
PAA_prom_rev	GTCATATTTGCCATCCATTTAATGGGGG GTTCTCCAGCCTTTTATGAAGG	Cloning *paaA* 5'-UTR into pUC18-mini-Tn7-Gm-lux
PAA_prom_seq_F	GAAGTTCCTATTCCGAAGTTC	Sequencing of pUC18-mini-Tn7-Gm-paa-lux
PAA_prom_seq_R	GCAGGTAAACACTATTATCACC	Sequencing of pUC18-mini-Tn7-Gm-paa-lux
PAA_prom_quik_F	GGCTGGAGAACCTGGATGGCAAATATG	Site directed mutagenesis of pUC18-mini-Tn7-Gm-paa-lux for optimised distance from RBS to start codon
PAA_prom_quik_r	CATATTTGCCATCCAGGTTCTCCAGCC	Site directed mutagenesis of pUC18-mini-Tn7-Gm-paa-lux for optimised distance from RBS to start codon
PAA_lux_F	CCACAGCGAATGGATAAGC	Screening of Ps652 clones with integrated pUC18-mini-Tn7-Gm-paa-lux
PAA_lux_R	CCAGATAATGGAACATTACCTGC	Screening of Ps652 clones with integrated pUC18-mini-Tn7-G-paa-lux

Table S3 Plasmids used in this study.

Plasmid	Application	Reference
pTS1	Suicide vector for in-frame deletions in *Pseudomonas*	6
pTS1-ΔHCN	In-frame deletion of *hcnABC* gene cluster in Ps652	This work
pTS1-Δ652Pyo	In-frame deletion of pyoverdine encoding NRPS in Ps652	This work
pTS1-ΔNRPS5	In-frame deletion of L-threonine encoding NRPS in Ps652	This work
pTS1-ΔtpoD	In-frame deletion of *tpoD* in Ps652 *tpo* BGC	This work
pTS1-ΔtpoE	In-frame deletion of *tpoE* in Ps652 *tpo* BGC	This work
pTS1-ΔtpoF	In-frame deletion of *tpoF* in Ps652 *tpo* BGC	This work
pTS1-ΔtpoG	In-frame deletion of *tpoG* in Ps652 *tpo* BGC	This work
pTS1-ΔtpoN	In-frame deletion of *tpoN* in Ps652 *tpo* BGC	This work
pME6032	Tetracycline-selective replicative plasmid for IPTG-inducible heterologous expression in *Pseudomonas*	7
pME6032-tpoD	Complementation of *tpoD* in Ps652Δ*hcn*Δ*tpoD*	This work
pUC18-mini-Tn7-Gm	Gentamicin-selective Tn7 integrative vector	8
pTNS2	Tn7 transposase expression to facilitate integration of mini-Tn7 vector.	8
pUC18-mini-Tn7-Gm-*tpoE*	Integrative Tn7 vector for complementation of Ps652 *tpoE* mutants	This work
pUC18-mini-Tn7-Gm-*tpoF*	Integrative Tn7 vector for complementation of Ps652 *tpoF* mutants	This work
pUC18-mini-Tn7-Gm-lux	Integrative Tn7 vector containing *luxCDABE* genes	9
pUC18-mini-Tn7-Gm-paa-lux	Integrative Tn7 vector containing *paaA* promoter linked to *luxCDABE* genes	This work
pET28b	Gene expression in *E. coli*	Novagen

Table S4 Media used in this study

Medium	Ingredient	Per Litre	Application
Lennox Broth and Lennox Agar (L)	Tryptone	10 g	Maintenance and growth of *Pseudomonas* strains
	Yeast extract	5 g	
	NaCl	5 g	
	D-glucose	1 g	
	Agar (Formedium)	10 g	
Lysogeny Broth (LB) or Lysogeny agar (LB agar)	Bacto-tryptone	10 g	Maintenance and growth of *E. coli*
	Yeast extract	5 g	
	NaCl	10 g	
	Agar (Formedium)	10 g	
Malt Extract-Yeast Extract Maltose Medium (MYM)	Malt extract	10 g	Cross-streak assays, split-plate assays
	Yeast extract	4 g	
	Maltose	4 g	
	Bacteriological agar	18 g	
	Tap water	1 L	
Rye Sucrose Agar (RSA)	Rye grains; pre-germinated, ground using stick blender, heated for 3 hours at 50 °C	60 g	Biological assays of Ps652 against *P. infestans*
	Sucrose	20 g	
Instant Mash Agar (IMA)	Instant mashed potato (Smash, Batchelors)	20 g	Biological assays of Ps652 fractions against *S. scabies* 87-22; growth of *S. scabies* 87-22
	Lab M #1 agar	20 g	
Modified King's Broth (MKB)	Glycerol	15 mL	5 L growth cultures for pyoverdine purification, Transposon mutant metabolomics
	K_2HPO_4 (anhydrous)	2.5 g	
	Casein hydrolysate	5 g	
	$MgSO_47H_2O$	2.5 g	
	Adjust to pH 6.7 with HCl		
Modified King's Broth + glucose (MKBG)	Glucose	15 g	Comparative metabolomics and for 2 L production of tropolones in Ps652 and mutants
	K_2HPO_4 (anhydrous)	2.5 g	
	Casein hydrolysate	5 g	
	$MgSO_47H_2O$	2.5 g	
	Adjust to pH 6.7 with HCl		
(CAS) agar	Prepared in accordance with ref. [10]		Iron binding assays

Table S5 Biosynthetic gene clusters in Ps652

Biosynthetic genes and clusters[1]	antiSMASH detection[2]	GECCO detection[3]	Biosynthetic class	From[4]	To[4]	Predicted product
1	No	No	Tropolone	380,822	396,119	3,7-dHT
2	Yes (1.1)	No	N-acetyl glutaminylglutamine amide (NAGGN)	937,812	952,547	NAGGN
3	Yes (1.2)	Yes	NRPS	1,443,088	1,528,685	Pyoverdine[5]
4	No	No	MddA-like methyltransferase[1]	1,961,558	1,962,307	Dimethyl sulfide (DMS)
5	Yes (1.3)	No	RiPP-like (YcaO)	2,951,482	2,963,686	Unknown peptide
6	Yes (1.4)	Yes	NRPS	3,015,864	3,055,397	Unknown peptide
7	No	Yes	DUF6388[5]	3,460,872	3,467,872	Unknown (few clear biosynthetic genes[6])
8	Yes (1.5)	Yes	NRPS	3,541,217	3,604,665	Pyoverdine[5]
9	Yes (1.6)	No	PKS-like (T3PKS or HMG-CoA synthase)	3,655,571	3,696,758	Unknown
10	No	Yes	PvcA-like isonitrile synthase[7]	4,444,442	4,452,702	Unknown
11	Yes (1.7)	Yes	RiPP-like	4,493,965	4,515,395	Quinohemoprotein amine dehydrogenase[8]
12	Yes (1.8)	Yes	T2PKS (aryl polyene)	5,056,186	5,099,787	Aryl polyene
13	Yes (1.9)	Yes	NRPS-like	5,244,418	5,287,864	*mgo*/*pvf*-like operon
14	No	No	HdtS-like acyl-homoserine lactone synthase[1]	5,373,110	5,373,880	*N*-acylhomoserine lactone
15	No	No	HCN	5,414,905	5,417,870	HCN
16	No	No	AldA-like indole-3-acetaldehyde dehydrogenase[1]	5,518,998	5,520,491	Indole-3-acetic acid (IAA)
17	Yes (1.10)	No	RiPP	5,884,355	5,906,514	pyrroloquinoline quinone (PQQ)

1. Genes predicted for the biosynthesis of DMS, *N*-acylhomoserine lactone and IAA are single genes rather than gene clusters. As these molecules can be defined as specialised metabolites, they are listed here.
2. antiSMASH 7.0.0 was used for analysis[11]. Numbers in parentheses refer to the antiSMASH annotation of the BGC. Note that antiSMASH 7.1.0 features rules for the detection of HCN BGCs.
3. https://gecco.embl.de/ v0.9.8 was used[12]. Where relevant, "Yes" indicates where GECCO BGC regions overlap with antiSMASH regions, although the precise boundaries are not identical.
4. antiSMASH or GECCO annotated boundaries are listed. Otherwise, manual annotations of BGC boundaries are based on the location of essential biosynthetic genes.
5. As in other *Pseudomonas* strains, the pyoverdine biosynthetic genes are located in two distinct loci.
6. DUF6388 proteins can be associated with phosphonate BGCs, but no other phosphonate genes are present in this putative cluster.
7. See ref. [13].
8. The quinohemoprotein amine dehydrogenase features a short RiPP peptide with three intrapeptidyl thioether cross-links introduced by a radical SAM protein[14,15].

Table S6 The tropolone biosynthetic gene cluster in Ps652

Protein Name	Size (AA)	Locus tag[a]	Protein ID[a]	Pfam domain(s)[b]	Predicted protein activity/domain	Validated function
TpoA	212	PS652_00387	CAK9887586.1	PF00440<br>PF17918	TetR family regulator	Positive regulator of biosynthesis in response to low iron[16].
TpoB	304	PS652_00386	CAK9887585.1	PF03328	Aldolase/citrate lyase family	
TpoC	246	PS652_00385	CAK9887584.1	PF13561	NAD(P)H-dependent reductase	
TpoD	137	PS652_00384	CAK9887583.1	PF13279	Acyl-CoA thioesterase	Essential for biosynthesis; biochemically characterised thioesterase activity.
TpoE	380	PS652_00383	CAK9887582.1	PF02770<br>PF00441<br>PF02771	Acyl-CoA dehydrogenase / epoxidase	Essential for biosynthesis; biochemically characterised.
TpoF	452	PS652_00382	CAK9887581.1	PF14535<br>PF00501	Phenylacetate-CoA ligase	Essential for biosynthesis. PAA-CoA production as pathway precursor.
TpoG	563	PS652_00381	CAK9887580.1	PF02776<br>PF00205<br>PF02775	TPP-dependent decarboxylase	Essential for biosynthesis; phenylacetate biosynthesis.
TpoH	264	PS652_00380	CAK9887579.1	PF13561	NAD(P)H-dependent reductase	
TpoI	488	PS652_00379	CAK9887578.1	PF02321	Outer membrane efflux protein	Transposon mutant disrupts biosynthesis[17].
TpoJ	360	PS652_00378	CAK9887577.1	PF00529	HlyD-like membrane-fusion protein	
TpoK	519	PS652_00377	CAK9887576.1	PF07690	Major facilitator superfamily	
TpoL	301	PS652_00376	CAK9887575.1	PF03466<br>PF00126	LysR family helix-turn-helix regulator	Positive regulator of biosynthesis in response to low iron[16].
TpoM	150	PS652_00375	CAK9887574.1	PF00582	Universal stress protein family	
TpoN	258	PS652_00374	CAK9887573.1	PF00378	Enoyl-CoA hydratase/isomerase	Essential for biosynthesis. Proposed role to intercept PAA catabolon for tropolone biosynthesis.

[a.] Locus tags and protein IDs are provided for the NCBI/ENA GenBank accession (OZ024668.1) of the *Pseudomonas* sp. Ps652 genome sequence.

[b.] Multiple Pfam domains are listed where they exist in different regions of each protein.

Table S7 Multistep gradient for semi-preparative scale polar C18 HPLC.

Retention Time (min)	% H_2O + 0.5% Formic Acid	% MeOH + 0.5% Formic Acid
0	100	0
5	100	0
6	85	15
30	50	50
35	0	100
40	0	100
41	100	0
46	100	0

FIGURES

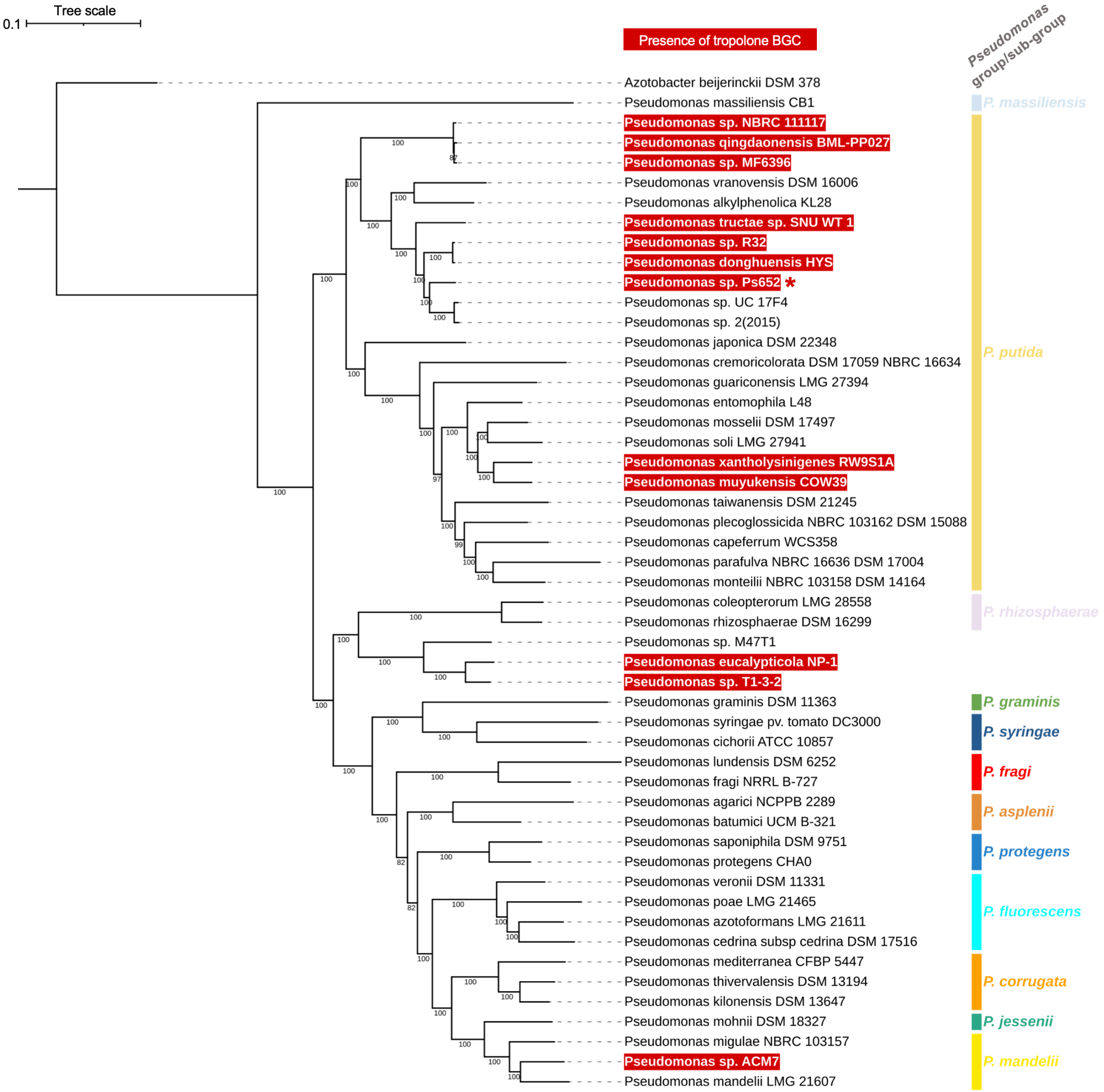

Figure S1 Phylogenetic analysis of Ps652 versus other *Pseudomonas* strains. Tree generated using AutoMLST[18] and visualised using iTOL[19]. AutoMLST identifies similar species to an input set of genomes to construct a tree featuring a total of 50 representative strains via a concatenated alignment of 83 conserved genes. Strains highlighted with a white text on a red background have a tropolone BGC, while *Pseudomonas* groups/subgroups described in Girard *et al.*[20] are highlighted by coloured strips. Girard *et al.* proposed that the strains within the Ps652 clade (including *P. donghuensis*, *P. tructae* and *P. vranovensis*) belong to the *P. vranovensis* sub-group. The clade containing *Pseudomonas eucalypticola* is not closely related enough (via average nucleotide identity) to *P. rhizosphaerae* to place these strains in the *P. rhizosphaerae* group so these strains are not associated with a specific group. Ps652 is highlighted with an asterisk.

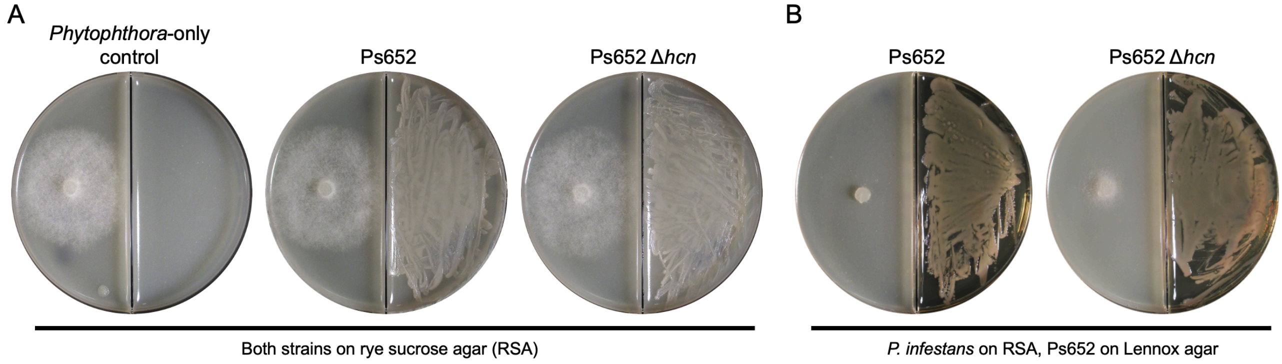

Figure S2 Split-plate assays for Ps652 and Ps652 Δ*hcn* versus *P. infestans*. **A.** Both pathogen and pseudomonad are plated on RSA. Little inhibition is observed, and no difference in inhibition is evident between the wild type Ps652 and the cyanide mutant Ps652 Δ*hcn*. **B.** Split-plate assays for Ps652 and Ps652 Δ*hcn* versus *P. infestans* with Ps652 strains plated on Lennox agar. Strong volatile production on Lennox agar provides complete inhibition of *P. infestans* by wild type Ps652. Some activity is lost in the cyanide mutant Ps652 Δ*hcn*, but significant inhibition of P. infestans remains, indicating at least one further volatile produced on Lennox agar. All images are representative of three biological replicates.

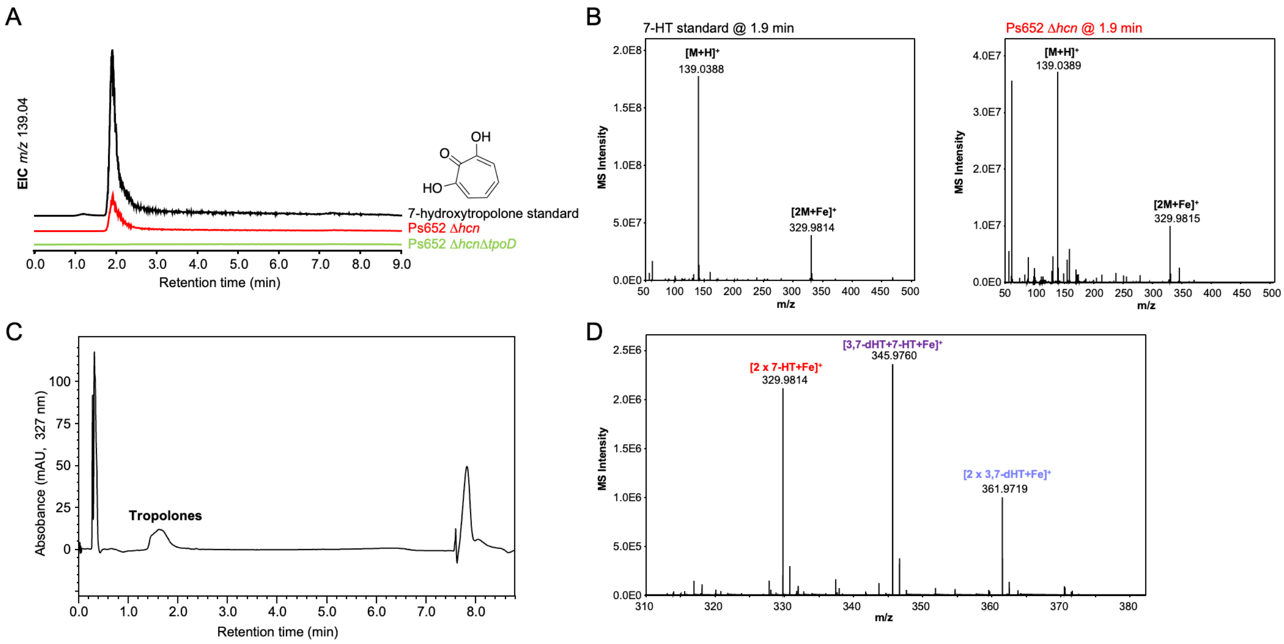

Figure S3 Detection of tropolones in Ps652. **A.** Production of 7-hydroxytropolone in Ps652 Δ*hcn* in comparison to a synthetic standard provided by Prof. Ryan Murelli (Brooklyn College, The City University of New York). Production is lost in the Δ*tpoD* mutant. EIC = extracted ion chromatogram. B. Comparison of MS spectra for standard 7-HT and the molecule produced in Ps652. C. UV chromatogram for the data shown in Figure 3B of the paper. D. MS spectrum showing the three $[2M+Fe]^+$ adducts of 7-HT and 3,7-dHT.

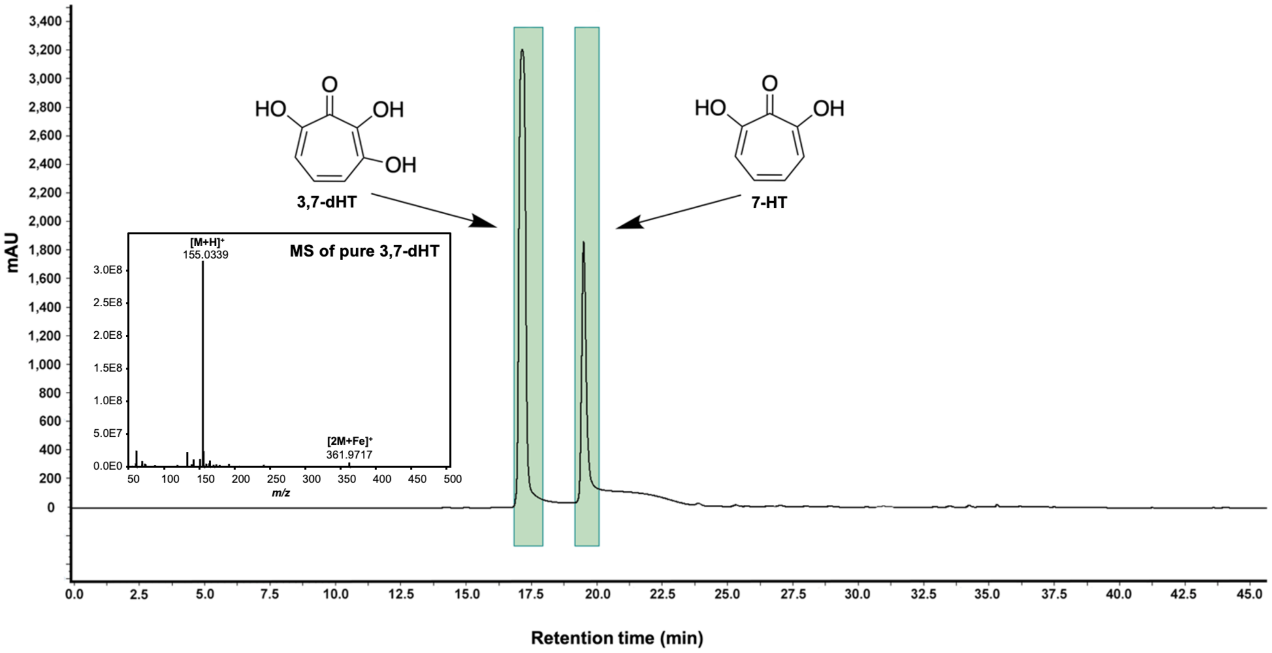

Figure S4 Separation of 7-HT and 3,7-dHT using semi-preparative HPLC. Trace shows UV absorbance at 327 nm. Inset, MS spectrum for purified 3,7-dHT.

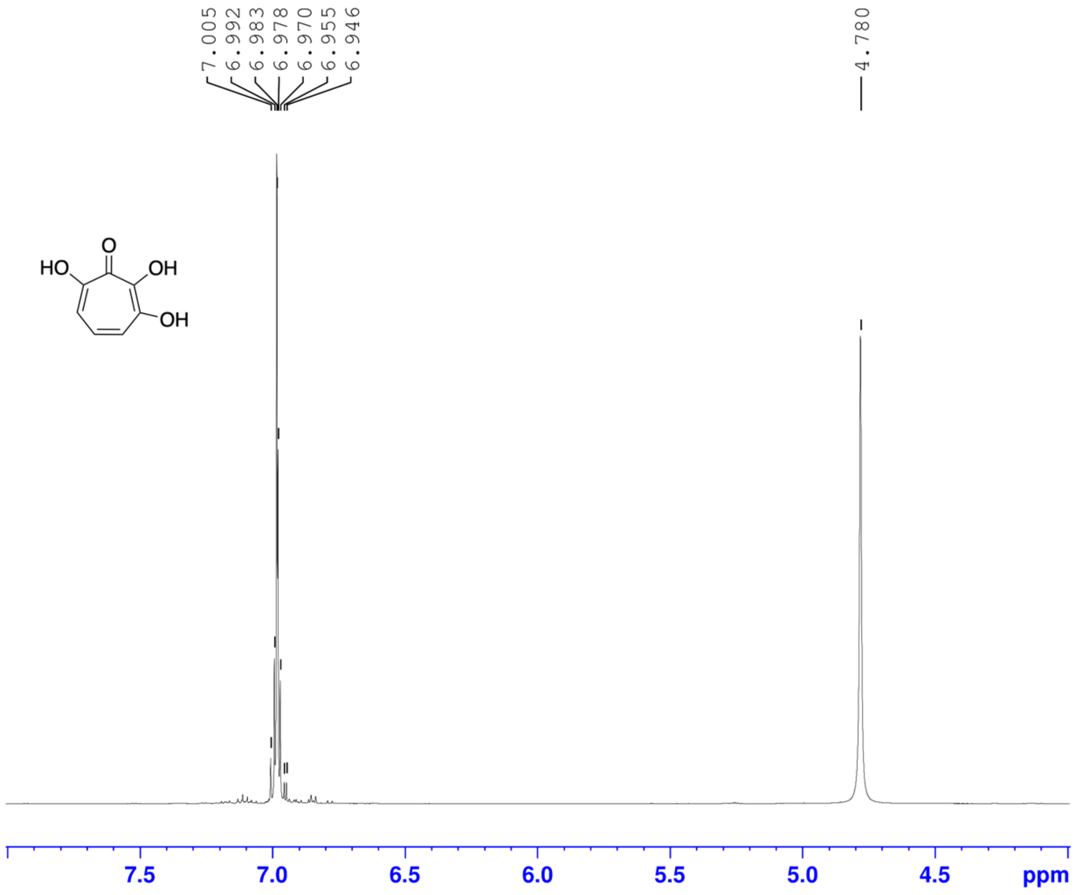

Figure S5 ^{1}H NMR spectrum (600 MHz, CD_3OD, 298 K) of 3,7-dHT. The multiplet at 6.98 ppm was identified as the 3 CH protons, which matches with to a previously published spectrum for this compound[21], where a singlet was observed due to the lower resolution instrument used for that study. The broad singlet peak at 4.78 ppm is the solvent peak.

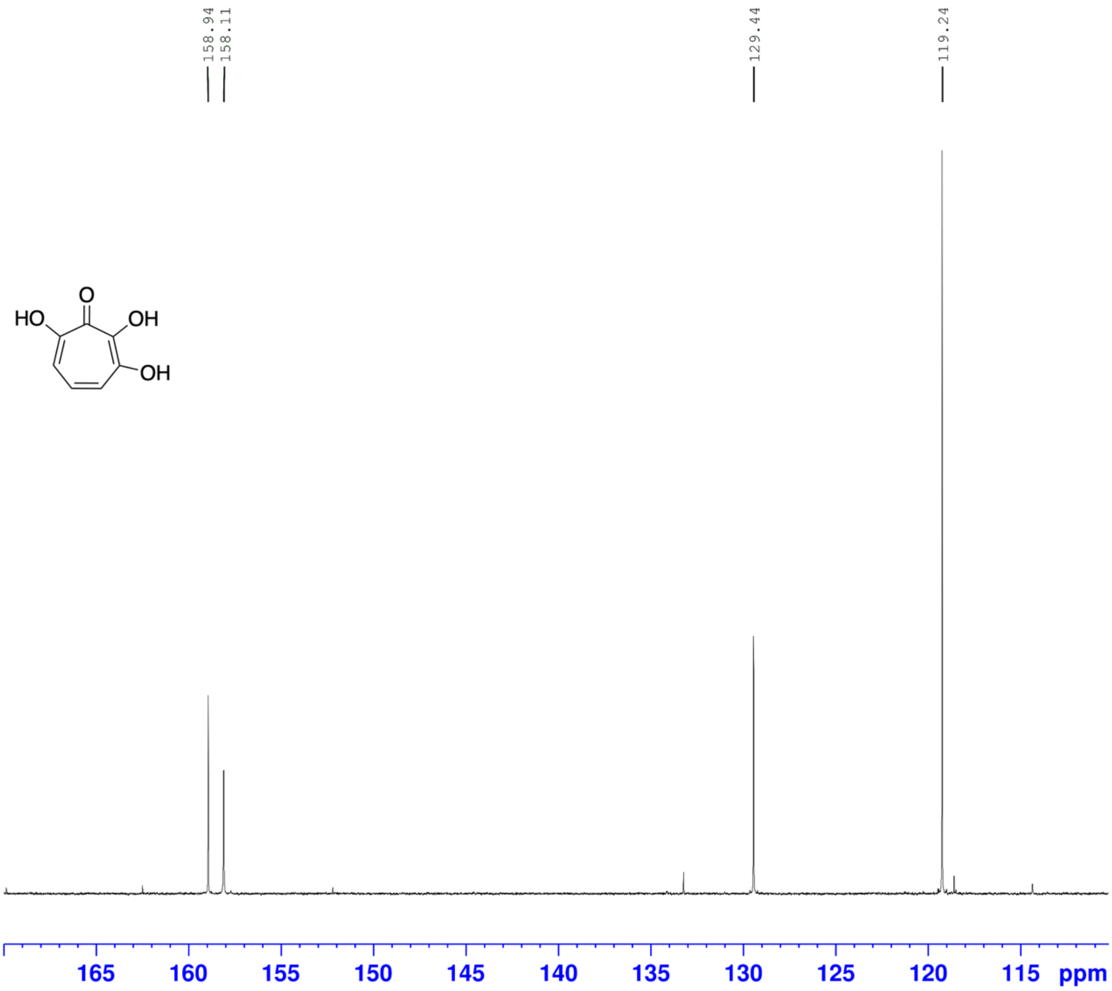

Figure S6 ^{13}C NMR spectrum (150 MHz, CD_3OD, 298 K) of 3,7-dHT.

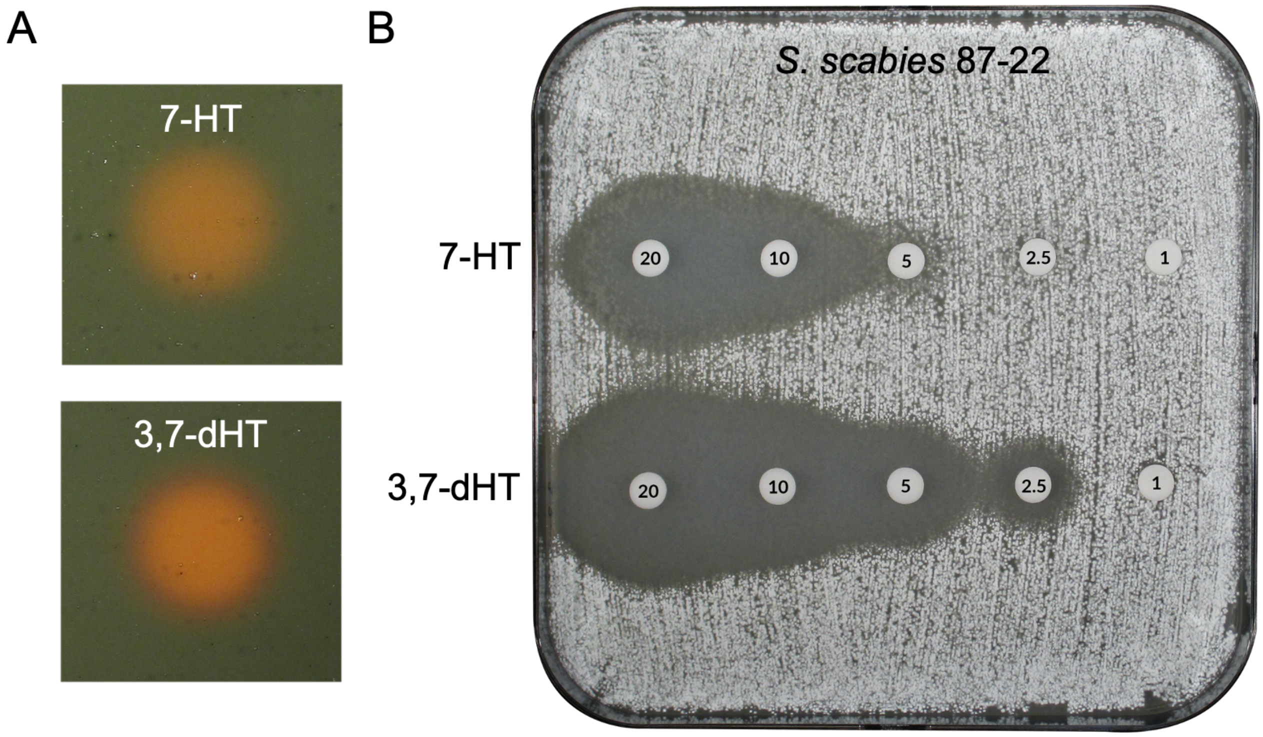

Figure S7 Comparison of antibacterial and iron binding activities of 7-HT and 3,7-dHT. **A.** Binding of 7-HT and 3,7-dHT to iron on chrome azurol S (CAS) agar plates. 5 µg each of 7-HT and 3,7-dHT were added to the surface of a CAS agar plate in 5 µL methanol. Both molecules displayed the ability to bind iron as indicated by the blue to orange colour change. B. Minimum inhibitory concentration (MIC) assay of 7-HT and 3,7-dHT with *S. scabies* 87-22. Numbers refer to micrograms of molecule in each paper disc.

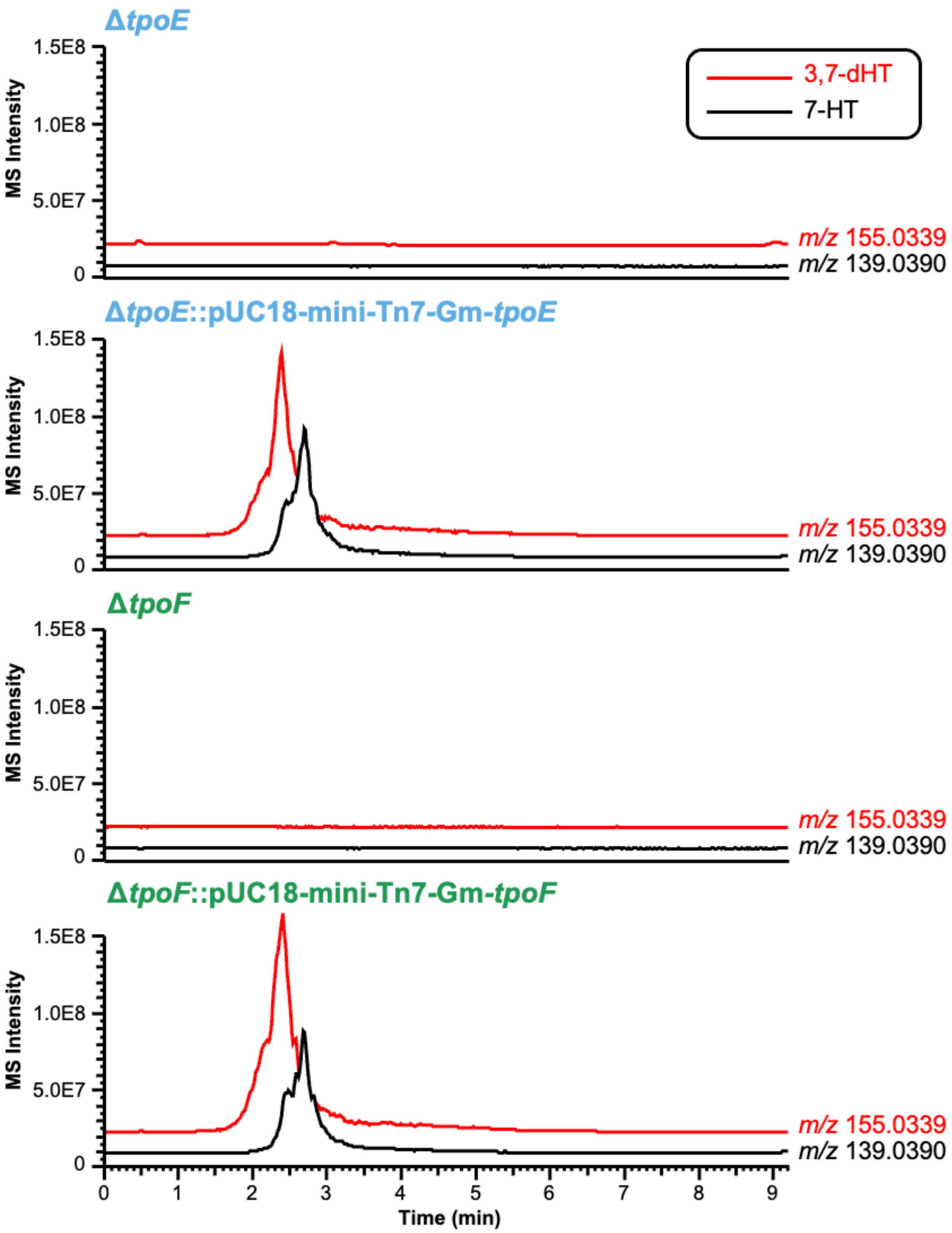

Figure S8 LC-MS chromatograms of Δ*tpoE* and Δ*tpoF* alongside strains complemented using pME6032-based plasmids. Each mass is searched within a ± *m/z* 0.007 window.

A

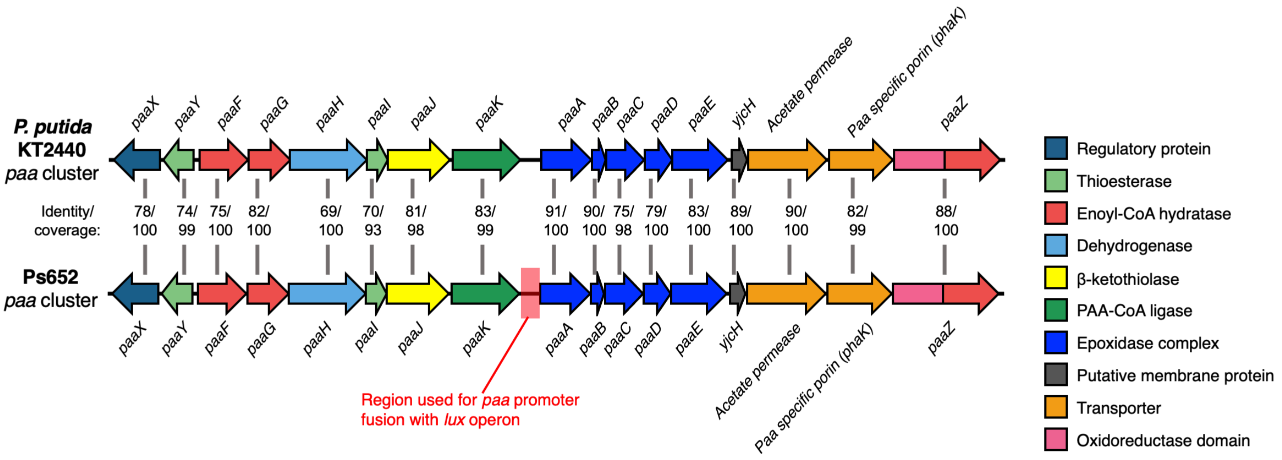

B

TTAGGTTCTTCAGGCCATCCCTGGGCCATGGGCCCTGGGATGGATGTTGAGGCTGCCAGCTTATCCATTCGTT
GTGGCGTGGCTGCCGACCCCTTCCGCCCTTACGGCGGCCTACTTTTCTCTTGGGAGGAAGTAGGCAAAACCGC
TTGCTCCTGTATACGGCCCTACGCTGCGCTTCGGGTCCCTTCGCTACGGCACCCCGCAAGAGCGCCACAGCGA
ATGGATAAGCGCTCCGTTACGCCCGAACAAAGCGATACACAAATCAGTTCTGATACACATTTTTGTGTTTGAT
ATTTTGCCCTGTATCACATATAACTGTCTCCAGCCTCAAAGCCCCCTTCATAAAAGGCTGGAGAACCCC

Figure S9 Overview of the PAA catabolon. A. Comparison of the PAA catabolon genes in *Pseudomonas putida* KT2440 and Ps652. The promoter region used for the *lux* reporter assays is highlighted in red. Where relevent, the colour coding for general protein activities is consistent with the *tpo* BGC. B. The sequence upstream of *paaA* in Ps652 used in the promoter fusion.

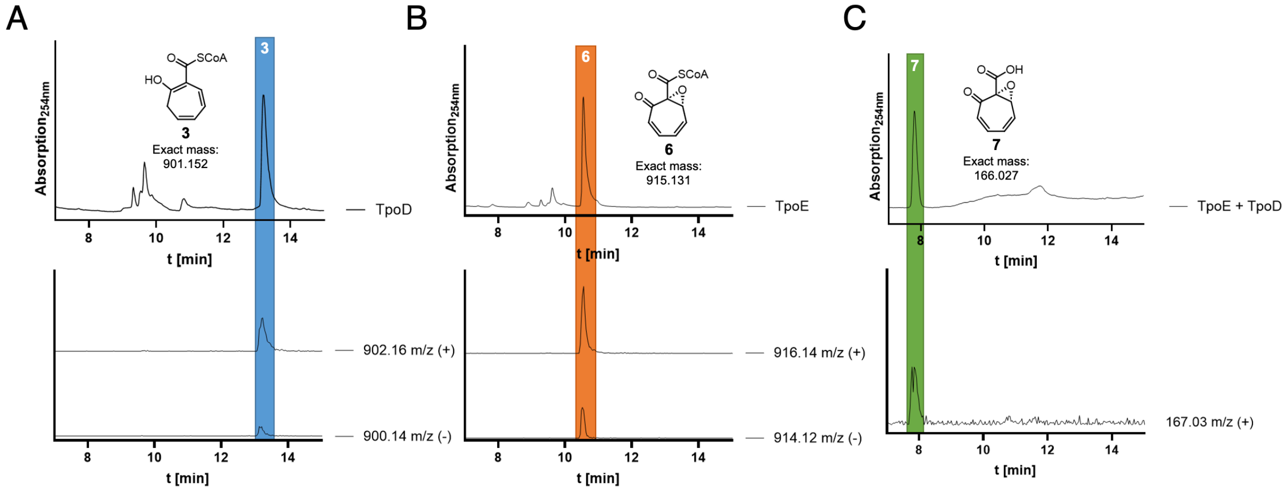

Figure S10 LC-MS analysis of TpoD, TpoE and TpoE + TpoD products from biochemical reactions. A. Positive and negative EICs of compound **3**. B. Positive and negative EICs for the TpoE product resulting from the conversion of **3**. C. Positive EIC for compound **7** generated by incubation with TpoE and TpoD (only positive mode shown due to poor ionization in negative mode).

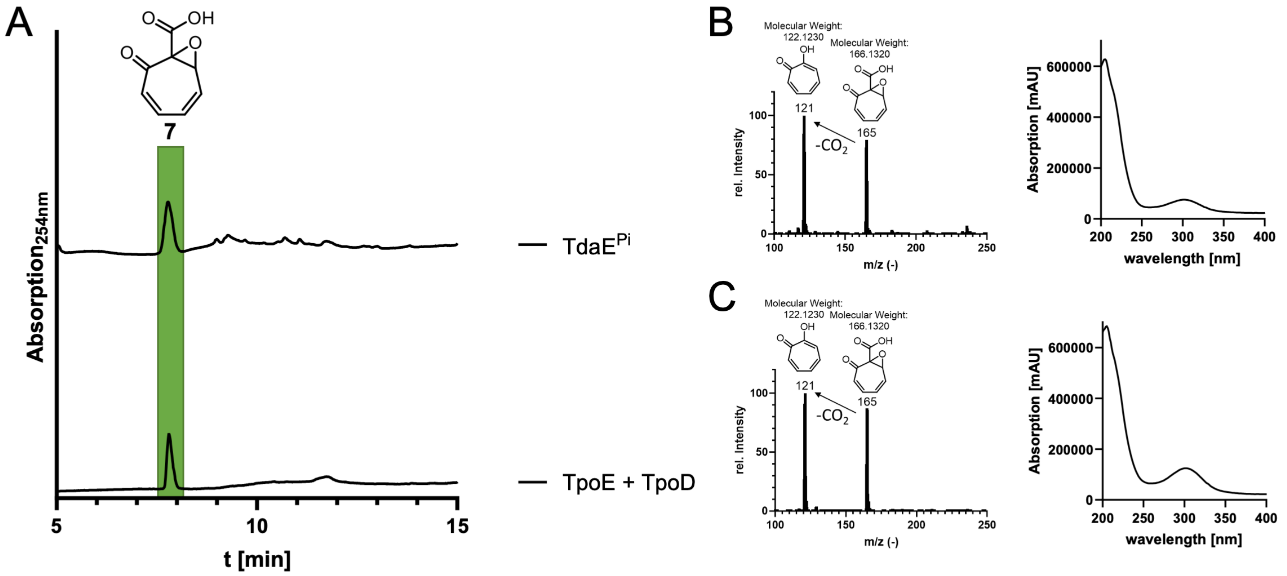

Figure S11 Comparison between compound **7** produced by $TdaE^{Pi}$ and TpoE + TpoD. $TdaE^{Pi}$ has previously been shown to catalyse the conversion of **3** into **7**.[22] A. HPLC analysis at 254 nm of compound **7** (highlighted in green) at 254 nm produced by TpoE + TpoD (bottom trace) or $TdaE^{Pi}$ (top trace). MS pattern with characteristic internal fragmentation (resulting from decarboxylation) and UV-visible spectrum of compound **7** produced by (**B**) $TdaE^{Pi}$ or (**C**) TpoE + TpoD.

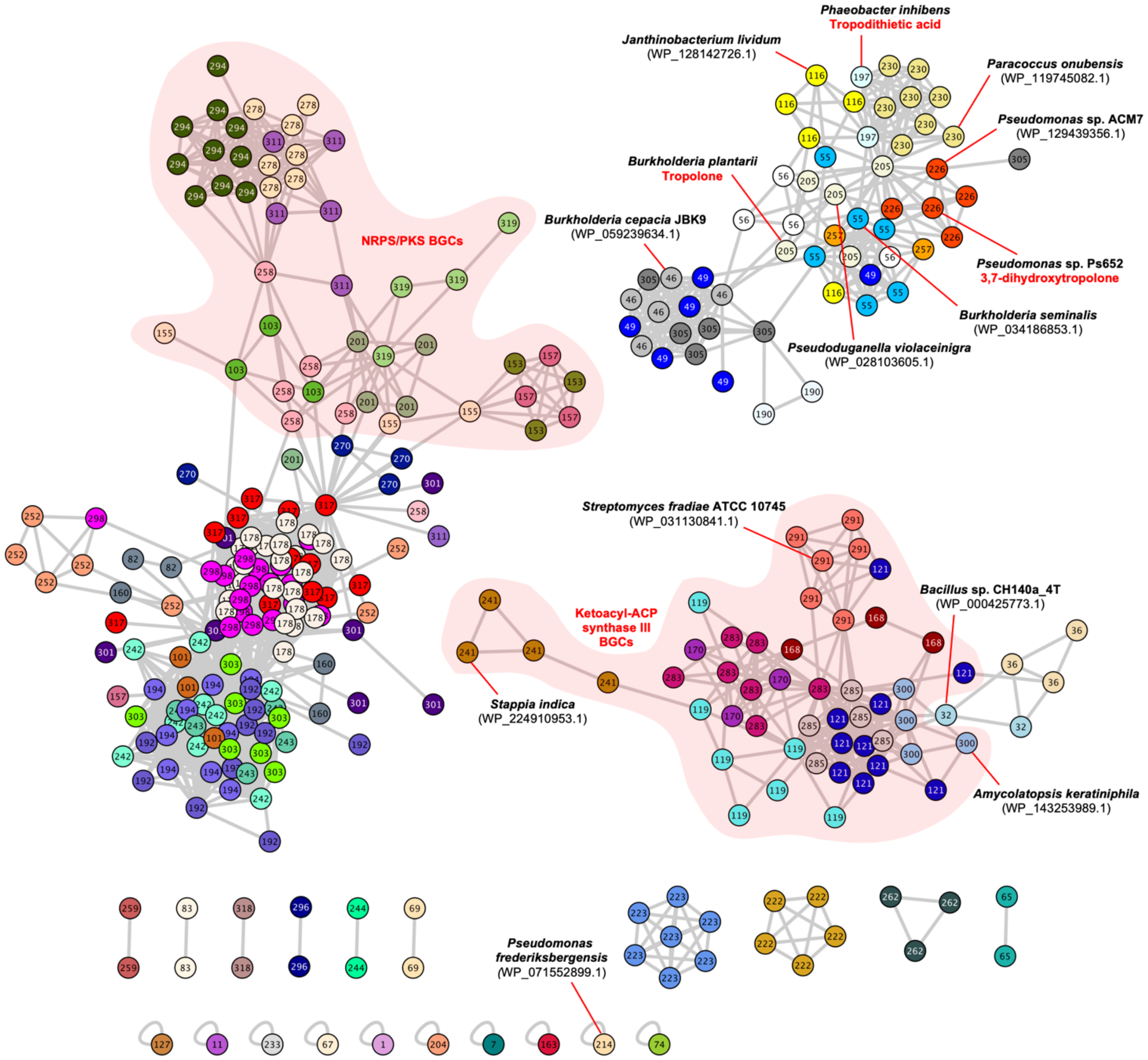

Figure S12 BiG-SCAPE[23] networking of BGCs retrieved from TpoE/TdaE-based genome mining. BGCs are labelled according to their family within the "Others" network with a 0.75 cut-off and are colour-coded to distinguish between families. The BGCs shown in Figure 6 are labelled, along with sets of BGCs that encode NRPS and/or PKS proteins alongside the flavoproteins (Figure S11). The BiG-SCAPE networks are visualised using Cytoscape.

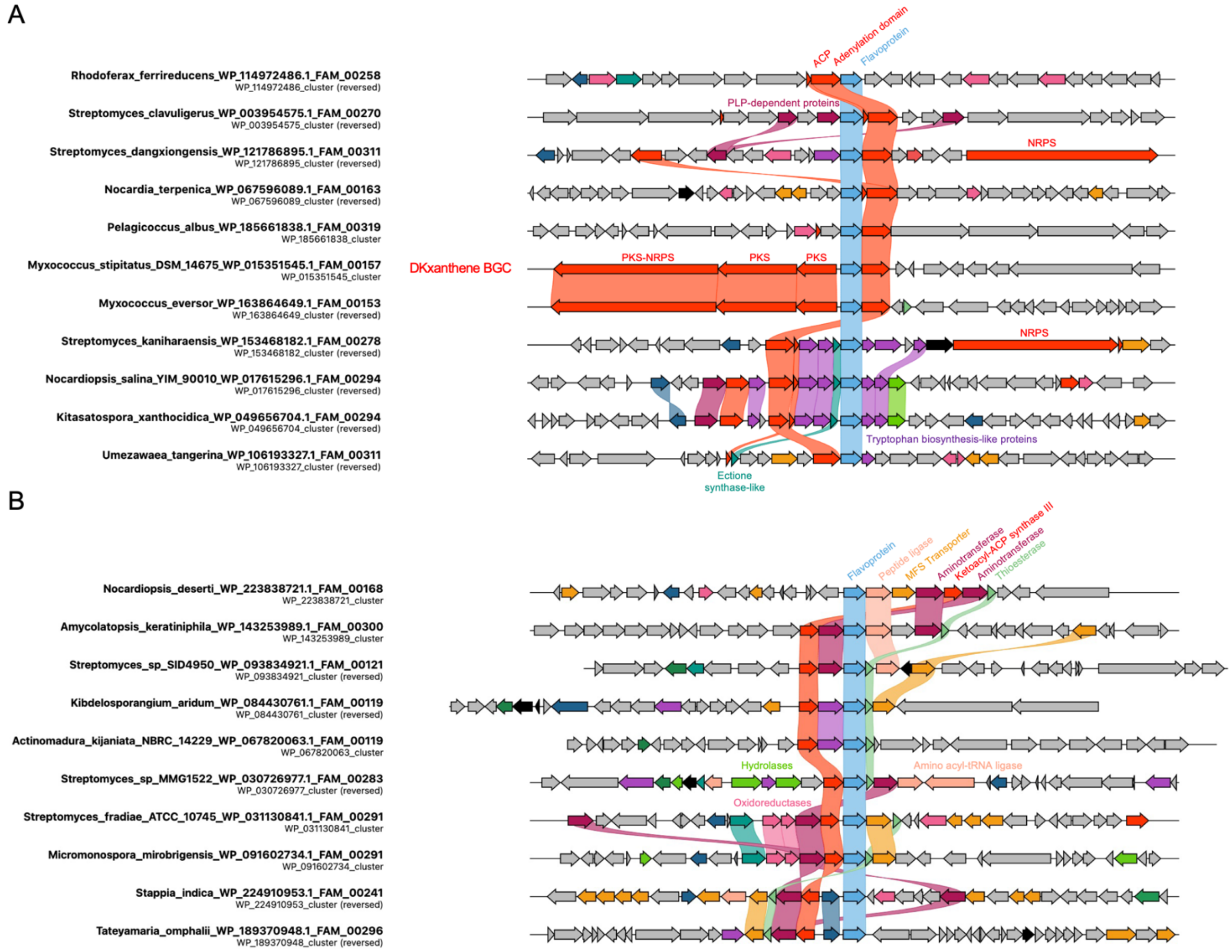

Figure S13 Examples of flavoprotein-associated BGCs that encode NRPS and/or PKS proteins. Family numbers, flavoprotein accession and species are listed with each BGC. Note that only genes with homology from the clinker analysis[24] (identity threshold of 30%) are colour-coded (see Supplementary File 2), so some BGCs do feature further assembly line genes, and some BGCs extend beyond the regions visible in the figure due to the criteria for BGC retrieval (35 kb regions centred on the flavoprotein gene). A. Example BGCs featuring NRPS or hybrid PKS-NRPS proteins. The BGC in *Myxococcus stipitatus* DSM 14675 is responsible for the production of DKxanthene[25] where it is proposed that the flavoprotein functions as a prolyl dehydrogenase[26]. B. Example BGCs that encode a conserved ketoacyl-ACP synthase III protein alongside multiple other conserved biosynthetic proteins, including a thioesterase.

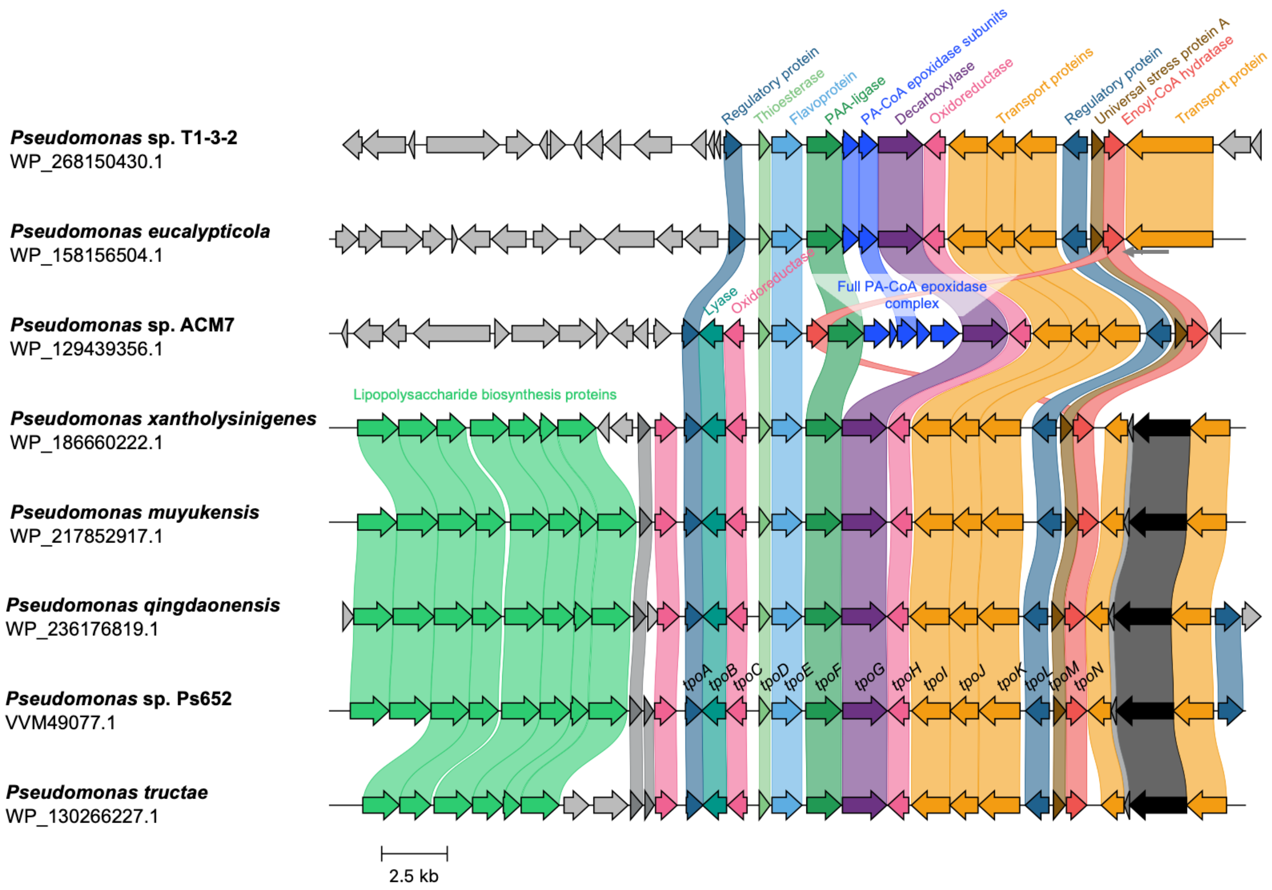

Figure S14 Comparison of tropolone BGCs in the genus *Pseudomonas*. These BGCs represent the diversity of BGCs with TpoE homologues with >50% identity. A 99% identity filter was used to reduce the redundancy of the dataset without limiting the diversity of output. A subset of BGCs encode some or all of the PA-CoA epoxidase subunits involved in the PAA catabolon, while *Pseudomonas* sp. ACM7 also has an extra copy of an enoyl-CoA hydratase gene. The *tpo* genes are listed for the Ps652 BGC and the accessions for the TpoE homologues are listed under each species name.

REFERENCES

1. Pacheco-Moreno, A., Stefanato, F. L., Ford, J. J., Trippel, C., Uszkoreit, S., Ferrafiat, L., Grenga, L., Dickens, R., Kelly, N., Kingdon, A. D., Ambrosetti, L., Nepogodiev, S. A., Findlay, K. C., Cheema, J., Trick, M., Chandra, G., Tomalin, G., Malone, J. G. & Truman, A. W. Pan-genome analysis identifies intersecting roles for Pseudomonas specialized metabolites in potato pathogen inhibition. *eLife* **10,** e71900 (2021).

2. Anton, B. P. & Raleigh, E. A. Complete Genome Sequence of NEB 5-alpha, a Derivative of Escherichia coli K-12 DH5α. *Genome Announc.* **4,** e01245-16 (2016).

3. Betancor, L., Fernández, M., Weissman, K. J. & Leadlay, P. F. Improved Catalytic Activity of a Purified Multienzyme from a Modular Polyketide Synthase after Coexpression with Streptomyces Chaperonins in Escherichia coli. *ChemBioChem* **9,** 2962–2966 (2008).

4. Loria, R., Bukhalid, R. A., Creath, R. A., Leiner, R. H., Olivier, M. & Steffens, J. C. Differential production of thaxtomins by pathogenic Streptomyces species in vitro. *Phytopathology* **85,** 537–541 (1995).

5. Kamoun, S., West, P. van, Vleeshouwers, V. G. A. A., Groot, K. E. de & Govers, F. Resistance of Nicotiana benthamiana to Phytophthora infestans Is Mediated by the Recognition of the Elicitor Protein INF1. *Plant Cell* **10,** 1413 (1998).

6. Scott, T. A., Heine, D., Qin, Z. & Wilkinson, B. An L-threonine transaldolase is required for L-threo-β-hydroxy-α-amino acid assembly during obafluorin biosynthesis. *Nat. Commun.* **8,** 15935 (2017).

7. Heeb, S., Itoh, Y., Nishijyo, T., Schnider, U., Keel, C., Wade, J., Walsh, U., O'Gara, F. & Haas, D. Small, stable shuttle vectors based on the minimal pVS1 replicon for use in gram-negative, plant-associated bacteria. *Mol. Plant-Microbe Interact.* **13,** 232–237 (2000).

8. Choi, K.-H., Gaynor, J. B., White, K. G., Lopez, C., Bosio, C. M., Karkhoff-Schweizer, R. R. & Schweizer, H. P. A Tn7-based broad-range bacterial cloning and expression system. *Nat. Meth.* **2,** 443–448 (2005).

9. Glassing, A. & Lewis, T. A. An improved Tn7-lux reporter for broad host range, chromosomally-integrated promoter fusions in Gram-negative bacteria. *J. Microbiol. Methods* **118,** 75–77 (2015).

10. Louden, B. C., Haarmann, D. & Lynne, A. M. Use of Blue Agar CAS Assay for Siderophore Detection. *J. Microbiol. Biol. Educ.* **12,** 51–53 (2011).

11. Blin, K., Shaw, S., Augustijn, H. E., Reitz, Z. L., Biermann, F., Alanjary, M., Fetter, A., Terlouw, B. R., Metcalf, W. W., Helfrich, E. J. N., van Wezel, G. P., Medema, M. H. & Weber, T. antiSMASH 7.0: new and improved predictions for detection, regulation, chemical structures and visualisation. *Nucleic Acids Res.* **51,** W46–W50 (2023).

12. Carroll, L. M., Larralde, M., Fleck, J. S., Ponnudurai, R., Milanese, A., Cappio, E. & Zeller, G. Accurate de novo identification of biosynthetic gene clusters with GECCO. *BioRxiv* 2021.05.03.442509 (2021). doi:10.1101/2021.05.03.442509

13. Clarke-Pearson, M. F. & Brady, S. F. Paerucumarin, a New Metabolite Produced by the pvc Gene Cluster from Pseudomonas aeruginosa. *J. Bacteriol.* **190,** 6927–6930 (2008).

14. Vandenberghe, I., Kim, J.-K., Devreese, B., Hacisalihoglu, A., Iwabuki, H., Okajima, T., Kuroda, S., Adachi, O., Jongejan, J. A., Duine, J. A., Tanizawa, K. & Beeumen, J. V. The Covalent Structure of the Small Subunit from Pseudomonas putida Amine Dehydrogenase Reveals the Presence of Three Novel Types of Internal Cross-linkages, All Involving Cysteine in a Thioether Bond. *J. Biol. Chem.* **276,** 42923–42931 (2001).

15. Nakai, T., Deguchi, T., Frébort, I., Tanizawa, K. & Okajima, T. Identification of Genes Essential for the Biogenesis of Quinohemoprotein Amine Dehydrogenase. *Biochemistry* **53,** 895–907 (2014).

16. Chen, M., Wang, P. & Xie, Z. A Complex Mechanism Involving LysR and TetR/AcrR That Regulates Iron Scavenger Biosynthesis in Pseudomonas donghuensis HYS. *J. Bacteriol.* **200,** 592 (2018).

17. Moffat, A. D., Elliston, A., Patron, N. J., Truman, A. W. & Lopez, J. A. C. A biofoundry workflow for the identification of genetic determinants of microbial growth inhibition. *Synth. Biol.* **6,** ysab004 (2021).

18. Alanjary, M., Steinke, K. & Ziemert, N. AutoMLST: an automated web server for generating multi-locus species trees highlighting natural product potential. *Nucleic Acids Res.* **47,** W276–W282 (2019).

19. Letunic, I. & Bork, P. Interactive Tree Of Life (iTOL) v5: an online tool for phylogenetic tree display and annotation. *Nucleic Acids Res.* **49,** W293–W296 (2021).

20. Girard, L., Lood, C., Höfte, M., Vandamme, P., Rokni-Zadeh, H., Noort, V. van, Lavigne, R. & Mot, R. D. The Ever-Expanding Pseudomonas Genus: Description of 43 New Species and Partition of the Pseudomonas putida Group. *Microorganisms* **9,** 1766 (2021).

21. Takeshita, H., Mori, A., Kusaba, T. & Watanabe, H. Preparation of Polyacetoxytropones and Polyhydroxytropolones by Acetolysis and Hydrolysis of Halotroponoids by Acetyl Trifluoroacetate with Exhaustive Displacement of Halogens on the Tropone Ring. Predominant Formation of Reductive Acetolysates from Fully-Substituted Tropones. *Bull. Chem. Soc. Jpn.* **60,** 4325–4333 (1987).

22. Duan, Y., Toplak, M., Hou, A., Brock, N. L., Dickschat, J. S. & Teufel, R. A Flavoprotein Dioxygenase Steers Bacterial Tropone Biosynthesis via Coenzyme A-Ester Oxygenolysis and Ring Epoxidation. *J. Am. Chem. Soc.* **143,** 10413–10421 (2021).

23. Navarro-Muñoz, J. C., Selem-Mojica, N., Mullowney, M. W., Kautsar, S. A., Tryon, J. H., Parkinson, E. I., Santos, E. L. C. de L., Yeong, M., Cruz-Morales, P., Abubucker, S., Roeters, A., Lokhorst, W., Fernandez-Guerra, A., Cappelini, L. T. D., Goering, A. W., Thomson, R. J., Metcalf, W. W., Kelleher, N. L., Barona-Gómez, F. & Medema, M. H. A computational framework to explore large-scale biosynthetic diversity. *Nat. Chem. Biol.* **16,** 60–68 (2020).

24. Gilchrist, C. L. M. & Chooi, Y.-H. clinker & clustermap.js: automatic generation of gene cluster comparison figures. *Bioinformatics* **37,** 2473–2475 (2021).

25. Hyun, H., Lee, S., Lee, J. S. & Cho, K. Genetic and functional analysis of the DKxanthene biosynthetic gene cluster from Myxococcusstipitatus DSM 14675. *J. Microbiol. Biotechnol.* **28,** 1068–1077 (2018).

26. Meiser, P., Weissman, K. J., Bode, H. B., Krug, D., Dickschat, J. S., Sandmann, A. & Müller, R. DKxanthene Biosynthesis—Understanding the Basis for Diversity-Oriented Synthesis in Myxobacterial Secondary Metabolism. *Chem. Biol.* **15,** 771–781 (2008).
